# Measurement of Ad Libitum Food Intake, Physical Activity, and Sedentary Time in Response to Overfeeding

**DOI:** 10.1371/journal.pone.0036225

**Published:** 2012-05-22

**Authors:** Jianying He, Susanne Votruba, Jeremy Pomeroy, Susan Bonfiglio, Jonathan Krakoff

**Affiliations:** Phoenix Epidemiology and Clinical Research Branch, Department of Health and Human Services, National Institute of Diabetes and Digestive and Kidney Diseases, National Institutes of Health, Phoenix, Arizona, United States of America; Institut Pluridisciplinaire Hubert Curien, France

## Abstract

Given the wide availability of highly palatable foods, overeating is common. Energy intake and metabolic responses to overfeeding may provide insights into weight gain prevention. We hypothesized a down-regulation in subsequent food intake and sedentary time, and up-regulation in non-exercise activity and core temperature in response to overfeeding in order to maintain body weight constant. In a monitored inpatient clinical research unit using a cross over study design, we investigated ad libitum energy intake (EI, using automated vending machines), core body temperature, and physical activity (using accelerometry) following a short term (3-day) weight maintaining (WM) vs overfeeding (OF) diet in healthy volunteers (n = 21, BMI, mean ± SD, 33.2±8.6 kg/m^2^, 73.6% male). During the ad libitum periods following the WM vs. OF diets, there was no significant difference in mean 3-d EI (4061±1084 vs. 3926±1284 kcal/day, p = 0.41), and there were also no differences either in core body temperature (37.0±0.2°C vs. 37.1±0.2°C, p = 0.75) or sedentary time (70.9±12.9 vs. 72.0±7.4%, p = 0.88). However, during OF (but not WM), sedentary time was positively associated with weight gain (r = 0.49, p = 0.05, adjusted for age, sex, and initial weight). In conclusion, short term overfeeding did not result in a decrease in subsequent ad libitum food intake or overall change in sedentary time although in secondary analysis sedentary time was associated with weight gain during OF. Beyond possible changes in sedentary time, there is minimal attempt to restore energy balance during or following short term overfeeding.

**Trial Registration:**

ClinicalTrials.gov NCT00342732

## Introduction

In developed countries, highly palatable foods are widely available and inexpensive, increasing the likelihood of overeating. However, despite our calorically dense, food rich environment which makes periods of overeating more common, susceptibility to weight gain varies. Thus, some physiological or behavioral regulation following or during these periods must exist to explain this variability. Immediate down regulation of food intake following overfeeding would be one possible mechanism to avoid weight gain. Yet it is unclear if this occurs in reality. In one previous study of young adults overfed for a longer period (13 days), subsequent ad libitum food intake was not decreased [1], [2] This lack of down regulation also raises the question of the importance of changes in appetite stimulating hormone such as ghrelin, and satiety hormones such as insulin, leptin, peptide tyrosine–tyrosine (PYY), glucagon-like peptide-1 (GLP-1) and their role in modulating food intake. In particular, for ghrelin, a putative hunger hormone, initial studies from our group demonstrated a negative association between total fasting ghrelin and ad libitum food intake [3]. However, follow-up studies in a larger cohort did not find any association between total fasting ghrelin concentrations and ad libitum food intake or that degree of suppression of fasting ghrelin following overconsumption from one day to the next predicted that days' food intake [4]. Thus, we investigated whether alternate mechanisms may play a role in resistance to weight gain during overconsumption.

Central changes in core body temperature which increase energy expenditure may reflect alterations in heat dissipation could be another mechanism by which weight gain is avoided during overfeeding. During weight maintenance, increased adiposity is associated with less abdominal and greater peripheral heat transfer [5]. Thus, differences in core body temperature reflecting differences in heat transfer may also be important during overfeeding.

As food intake may not precisely defend against overeating, changes in physical activity has been proposed as an alternative mechanism to maintaining weight. Levine et al found that increased non-exercise physical activity thermogenesis (NEAT), which describes physical activities of daily living including fidgeting, maintenance of posture and other physical activities except purposeful exercise, defends against fat gain in response to overfeeding [6]. Whether non-exercise activity truly changes with overfeeding is controversial [7]. Even so, in response to chronic positive energy balance, NEAT may be one mechanism which acutely protects against weight gain during overeating.

While many overfeeding studies have examined adaptive mechanisms, they have done so over longer (several weeks) or very short (meal to meal) periods and have tended to concentrate on one part of the energy balance equation [8], [9]. Periods of overeating and subsequent caloric compensation are more likely to occur over more defined periods. Therefore, in a controlled inpatient setting using a crossover design, we investigated changes in ad libitum food intake, core body temperature, non-exercise activity, and hormones possibly related to food intake following short term (3 day) overfeeding.

## Methods

### Subjects

Thirty-one volunteers were screened for this study from October 2007 to July 2009. Eight subjects were excluded for not meeting the criteria and other reasons. Twenty-three volunteers without diabetes and without a history of eating disorders were included in this study. Volunteers were recruited from newspaper advertisements and local flyers. Written informed consent was obtained and study details were explained to all volunteers before admission. All volunteers were found to be free of disease according to physical examination, medical history, and laboratory tests. Volunteers were admitted to the Clinical Research Unit of the National Institute of Diabetes and Digestive and Kidney Diseases (NIDDK) in Phoenix, Arizona, for approximately 20 days. The unit is housed on a single floor and has an area of approximately 7500 square feet. Subjects could walk freely on the unit, but were instructed not to exercise. There is a room with a pool table and subjects could go off the unit approximately 3 times a week for a supervised 30 minute outing. The study was approved by the NIDDK Institutional Review Board.

### Study design

The protocol for this trial and supporting CONSORT checklist and flow chart are available as supporting information; see Checklist S1, Flowchart S1 and Protocol S1.

**Figure 1:**
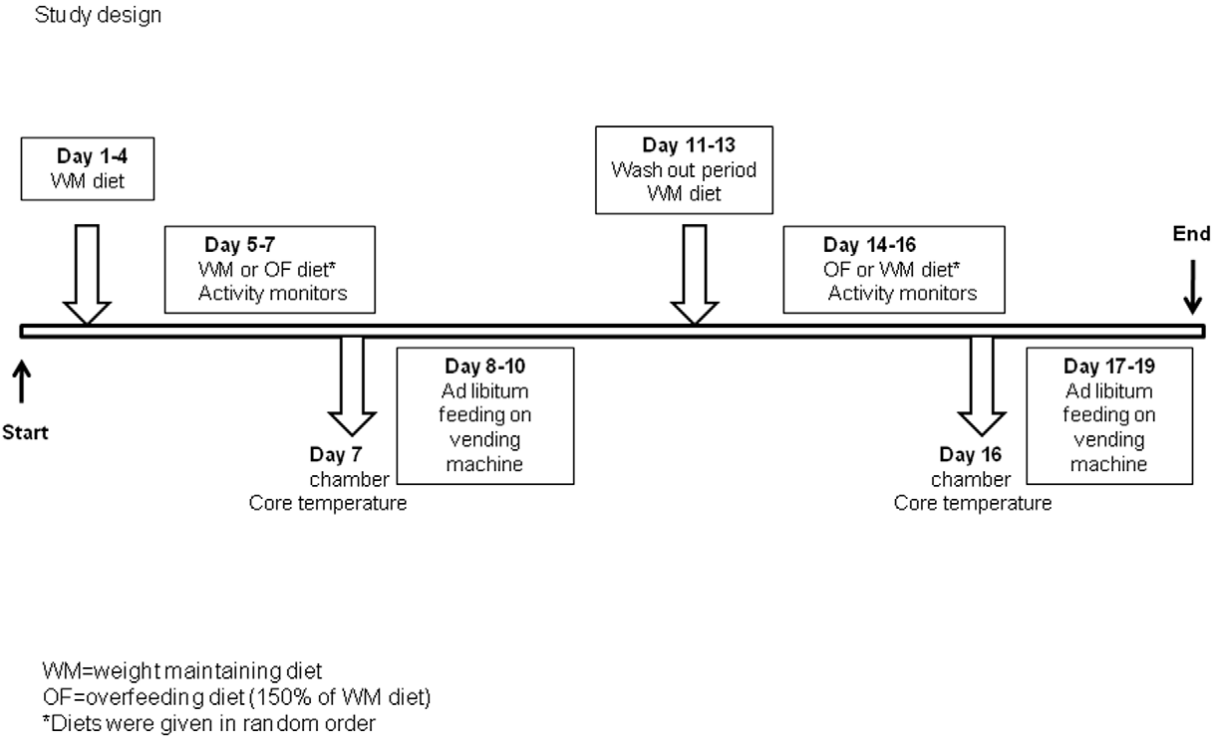
On admission volunteers were started on a standard weight maintaining diet for 4 days. On days 5–7 volunteers were randomized to continue on their weight maintaining diet (WM) for an additional 3 days or start 3 days of an overfeeding diet (OF) equal to 150% of their weight maintenance diet in calories. During each 3 day diet period volunteers also wore accelerometers. On the final day of the WM or OF periods, volunteers were placed in the respiratory chamber for 24 hours for measurement of energy expenditure and they received the core temperature capsule. On days 8–10, ad libitum food was assessed using the automated vending machine. Following the 3 days of ad libitum food intake, volunteers resumed their weight maintaining diet for 3 days (as a wash out period) followed by another 3 days of either the WM or OF diet and once again followed by 3 days of ad libitum food intake using the vending machines.

The study sample size (n = 30) was modeled on the initial analysis by Salbe et al [3]. However, after analysis by Votruba et al failed to find an association between suppression of fasting ghrelin concentrations following overconsumption and ad libitum food intake [4]. The current analysis focuses on the difference in ad libitum food intake following WT and OF diet. The sample size estimate based on the data from our previous vending studies was as follows: with a sample size of 20 subjects, we had a power of 0.92 at an alpha of 0.05 to see a 10% reduction in food intake after overfeeding. Therefore, we elected to perform an analysis of our collected data on food intake including changes in activity, and core body temperature. On admission volunteers were started on a standard weight maintaining diet (20% protein, 30% fat, 50% carbohydrate) for 4 days (Figure 1). Height was measured with a stadiometer; weight was measured by an electronic digital scale. Weight-maintenance energy needs (WMEN) specific to the inpatient unit were calculated based on body weight and sex(10). On the second day after admission, body composition was assessed by using dual energy X-ray absorptiometry (DXA; Lunar Corp, Madison, WI) and percentage body fat (%BF), fat mass (FM), and fat-free mass (FFM) were calculated as previously described [10]. On day 4, a 75g oral glucose tolerance test was performed with insulin and glucose concentrations measured at −15, 0, 30, 60, 120 and 180 minutes. On days 5–7 volunteers were randomized by 2×2 crossover design to continue on their weight maintaining diet (WM) for an additional 3 days or start 3 days of an overfeeding diet (OF) equal to 150% of their weight maintenance diet in calories.

During each 3 day diet period volunteers also wore accelerometers on each wrist, ankle and at the waist. Volunteers were required to consume all their food. On the final day of the WM or OF periods, volunteers were placed in the respiratory chamber for 24 hours for measurement of energy expenditure and they received the core temperature capsule. On days 8–10, ad libitum food was assessed using the automated vending machine. Following the 3 days of ad libitum food intake, volunteers resumed their weight maintaining diet for 3 days (as a wash out period) followed by another 3 days of either the WM or OF diet and once again followed by 3 days of ad libitum food intake using the vending machines.

### Food intake evaluation

For the measurement of ad libitum food intake, automated vending machines were used as previously described [3], [11]. In brief, the foodstuffs made available to the volunteers in the automated food-vending systems were based on a food preference questionnaire [12]. For each day on the vending system, the same 40 food items as well as a core group of condiments, juice, and soda were provided. Each volunteer was assigned to a single, refrigerated vending machine and had access to the machine for 23.5 hours each day. Food selection was monitored by the computerized vending system, and food wrappers and unconsumed food were returned to the metabolic kitchen to be weighed. Energy and macronutrient intakes during the ad libitum feeding period were calculated using the Food Processor Professional Diet Analyzer Program (ESHA version 10.0.0, ESHA Research, Salem, OR) as previously described [13]. All volunteers spent 3 days selecting foods from the vending machine and results are presented as the mean daily energy intake (DEI) over that period. Percent WMEN (%WMEN) was calculated by DEI/WMEN*100%.

### Non-exercise activity and sedentary time measurement

During the stay in the research unit, no exercise was performed for each subject. Non-exercise activity and sedentary time were measured with an omnidirectional, uni-axial accelerometer (Actical, Philips Respironics, Bend, OR, USA). Activity is expressed by the Actical in device-specific arbitrary units (counts per minute). Subjects were asked to simultaneously wear five Actical monitors for the 3 day period of each WM or OF period that preceded the ad libitum food intake. An Actical monitor was worn at the waist, as well as on each wrist and ankle. Each Actical was set to record in 1 minute epochs. Data was downloaded from the monitors using the manufacturer's software. Additional processing to calculate the non-exercise activity and sedentary time variables was accomplished using programs written in SAS. Sedentary time was calculated as the percentage of wear time where activity counts were less than 10 per minute [14]. Sedentary time was estimated using only the waist-worn monitor. Non-exercise activity (expressed as average counts per minute) was calculated by averaging the counts per minute during periods when the monitor was worn for each of the Actical monitors.

### Sedentary 24 hour and sleep energy expenditure measurement

Sleeping and 24 hour energy expenditure were measured in a respiratory chamber as previously described [15]. Briefly, volunteers entered the respiratory chamber at 0800 after eating breakfast and remained there for 23.25 h. Because of confinement within the chamber, calories fed to volunteers during the WM and OF diets were approximately 80% of that consumed while on the research unit. Oxygen and carbon dioxide concentrations were measured using a Siemens analyzer (OXYMAT 6; Siemens GmbH, Karlsruhe, Germany) and ABB analyzer (AO 20∶20; ABB Automation, GmbH, Frankfurt am Main, Germany); O_2_ and CO_2_ concentrations from the last 8 seconds of each minute were used to calculate the amount of VO_2_ consumed and VCO_2_ produced as previously described [16]. Spontaneous physical activity (SPA) was detected by radar sensors and expressed as the percentage of time over the 24 h period in which activity was detected. Sleeping metabolic rate was defined as the average energy expenditure between 2330 and 0500 during which SPAwas <1.5%. Propane burn tests to determine the accuracy of the energy expenditure measurement demonstrated mean recoveries of ±1% for O_2_ and CO_2_.

### Core body temperature measurement

Core body temperature was measured via an ingested capsule (HQ Inc, Palmetto, Florida) through which a wireless temperature signal was sent to a wireless monitor worn on the volunteers' hip. Volunteers swallowed the capsule at 5AM on the morning of the planned metabolic chamber. Prior to ingestion, the temperature capsule was calibrated to a standard mercury thermometer. Capsules which varied from the standard thermometer by >0.5°C were discarded. Linear regression was used to correct the core temperature capsule to the standard mercury thermometer. The accuracy of core temperature was to ±0.2°C. Room temperature in the respiratory chamber was maintained at close to 23.0°C when core temperature was measured on both WM and OF. Data was downloaded via the program provided by (HQ Inc, Palmetto, Florida). The interval for each core temperature reading was 1 min.

### Hormone measurements

GLP-1, PYY and leptin were measured using the Milliplex Human Gut Hormone panel kit from Millipore (Billerica, MA). This panel developed for the Luminex xMAP platform simultaneously measures GLP-1, PYY and leptin. The intra-assay variation (%CV) was <11% and the inter-assay variation (%CV) was <19%.

### Plasma glucose and insulin measurement

Plasma glucose concentration was determined by the glucose oxidase method (Beckman Instruments, Fullerton, CA). Plasma insulin concentrations were measured by AT AIA Pack IRI on TOSOH analyzer (Tosoh Bioscience, King of Prussia, PA).

### Statistical Analysis

Statistical analyses were performed using SAS software (SAS version 9.1, SAS Institute, Inc., Cary, NC). Normality of the data was tested by the Shapiro-Wilk test. Non-normally distributed variables were log transformed to approximate normal distributions. If normal distribution was not achieved by logarithmic transformation, nonparametric tests were used. Student's t-test and Wilcoxon test was used for sex comparisons of continuous variables. Paired t-tests were used to compare variables measured on each diet. Pearson or Spearman correlation analysis was used to test the relationships between continuous variables. Pearson analysis was used for the correlation of sedentary time with weight change, A-ghrelin with subsequent food intake, and r was used to designate Pearson correlation as correlation coefficient. Spearman correlation analysis was used for T-ghrelin, Leptin, GLP-1, PYY with subsequent food intake, as T-ghrelin, Leptin, GLP-1 and PYY did not reach a normal distribution when log10 transformed, and rho was used to designate Spearman correlation. ANOVA was used to test the difference in food intake following OF for 3 days. As noted above, for each capsule the core temperature was corrected to a standard mercury thermometer based on calibration measurements performed the night prior to ingestion using general linear regression models. All data are presented as mean±SD unless stated otherwise, and p<0.05 is taken as significant.

## Results

### Subject Characteristics

Subject anthropometric and insulin measurements are shown in table 1. In table 1, there were 13 volunteers with normal glucose regulation status (NGR) with a fasting glucose<100 mg/dl and 2 h glucose <140 mg/dl and 8 with impaired glucose regulation status (IGR) with 100 mg/dl = <fasting glucose<126 mg/dl and 140 mg/dl = <2 h glucose <200 mg/dl. Volunteers were unaware of their glucose regulation status until the end of the study. Energy intake, macronutrient intake, macronutrient composition, 24EE and Sleep EE, energy balance, sedentary time and spontaneous physical activity are shown in table 2. The macronutrient composition of the OF diet was the same as the WM diet.

**Table 1:** Subject characteristics.

**N (M/F)**	21 (15/6)
**Age**	42 (9)
**BMI (kg/m^2^)**	33.2 (6.5)
**%BF**	34.6 (10.5)
**Fasting glucose (mg/dl)**	95.4 (6.0)
**2 h glucose (mg/dl)**	125.1 (35.2)
**Fasting insulin (µu/ml)**	11 (7–22)
**2 h insulin (µu/ml)**	46 (19–106)
**NGR/IGR**	13/8
**Weight change by WM (kg)**	−0.11 (0.69)
**Weight change by OF (kg)**	0.67 (0.98)

M = Males, F = females; NGR, normal glucose regulation status; IGR, impaired glucose regulation status. Data are means (SD) or median (25%–75%) percentile; Weight change calculated as the difference of morning body weight between the next day finishing WT or OF diet and the day starting WT or OF diet.

**Table 2:** Energy and Macronutrient Intake, Energy Expenditure and Non-exercise activity.

	WT diet	Ad libitum following WT	OF diet	Ad libitum following OF
**Energy intake (kcal/d)**	2810	4061 (1083)	4215	3926 (1284)
**Percent of energy intake by weight Maintenance energy needs (%)**	100	145	150	140
**Macronutrient composition Carbohydrate, Fat and Protein (%)**	50, 30, 20	47, 40, 13	50, 30, 20	48, 39, 13
**24EE (kcal/d)**	2355.3 (311)	-	2588.7 (290)	-
**Sleep EE (kcal/d)**	1893.4 (283)	-	2066.7 (296)	-
**Energy balance (kcal/d)**	−102 (311)	-	784 (290)	-
**Weight change (D2-D1)** *	0.11 (0.36)	-	0.44 (0.54)	-
**Weight change (D3-D2)** *	0.03 (0.47)	-	0.38 (0.41)	-
**Sedentary time in unit (%)**	70.9 (12.9)	-	72 (7.4)	-
**Sedentary time in chamber (%)** **	74.4(6.6)	-	78.4 (6.6)	-
**Waist Non-exercise activity by counts (arbitrary unit)**	93.9(21.5)	-	68 (18.4)	-
**Spontaneous physical activity (%)**	5.6 (5.0–6.5)	-	5.5 (4.9–6.9)	-

Data are means (SD) or median (25%–75%) percentile. D1, D2 and D3, day 1, day 2 and day3 on WT or OF diet.

** P<0.01,

* P<0.05,

P values are analyzed using paired T-test to compare WT and OF diets.

### Comparison of mean daily energy and macronutrient intake following the weight maintenance and overfeeding diets

**Figure 2:**
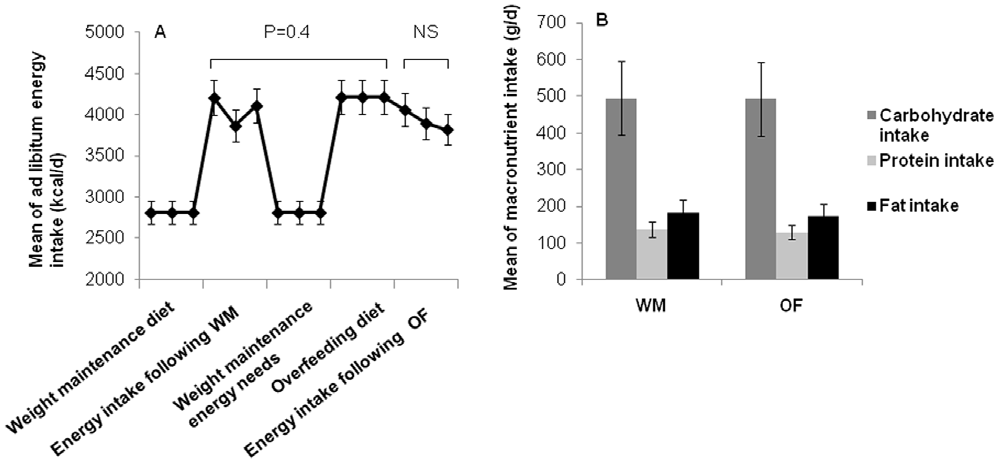
Mean daily energy and macronutrient intake following weight maintenance diet (WM) and overfeeding diet (OF). Fig. 2A. Daily energy intake during the study for each day. Ad libitum mean daily energy intake was 4061±1084 (kcal/d) following WM, and 3926±1284 (kcal/d) following OF. There was no difference in mean of daily energy intake (p = 0.4) between WM vs. OF. A decline in energy intake over 3 day ad libitum food intake period following OF is noted, but the trend was not significant (p = 0.9). Fig. 2B. Mean of daily carbohydrate, protein, and fat intake were 484±126 (g/d), 138±42 (g/d), 183±61 (g/d) following WM, and 477±156 (g/d), 129±45 (g/d), and 173±74 (g/d) following OF. No difference was found in carbohydrate (p = 0.7), protein (p = 0.2) or fat (p = 0.3) intake between WM vs. OF. All data are means ± SD. Paired t-test was used to analyze differences between diets.

Twelve subjects received the OF diet first, but only 10 were included in the analysis as 2 were found to be sharing food; 11 received the WM diet first. Overall, men had higher mean daily energy (p<0.01) and carbohydrate intakes (p<0.01) compared with women. Mean 3 day daily ad libitum energy intake (calculated as mean kcal/day) was not different following WM compared to OF (4061±1083 vs. 3926±1284 (kcal/d; p = 0.4)(Figure 2A), nor were any of the macronutrient intakes different (Figure 2B). There was a trend toward a slight but non-significant decrease (p = 0.9) in food intake over the 3 day ad libitum period following OF. Diet order did not affect any of the energy intake measures, nor did glucose regulation status.

As noted in table 3, hormone concentrations before and after both WM and OF were not significantly different. The fasting hormone concentrations at the start of the ad libitum periods following either the WM or OF diets were not associated with subsequent food intake (Table S1). In the case of ghrelin, this was consistent with results we have previously reported [4].

**Table 3:** Fasting Circulating Hormones Concentrations prior to and following each Diet.

	Before	After	P
**A-ghrelin (pg/ml) (WM)**	32.3 (18.7)	28.4 (19.6)	
**A-ghrelin (pg/ml) (OF)**	27.3 (14.7)	28.1 (20.4)	0.5
**T-ghrelin (pg/ml) (WM)**	102.2 (49.3–187.5)	120.2 (40.9–218.4)	
**T-ghrelin (pg/ml) (OF)**	94.1 (48.9–136.7)	123.4 (43.0–159.0)	0.3
**Leptin (ng/ml) (WM)**	10.4 (4866.6–22445.9)	8.6(4872.0–17028.4)	
**Leptin (ng/ml) (OF)**	10.6 (4978.8–22808.2)	9.7 (4999.4–21664.7)	0.5
**GLP-1 (pg/ml) (WM)**	14.1(11.5–18.9)	13.7 (10.6–21.7)	
**GLP-1 (pg/ml) (OF)**	14.6(7.0–22.2)	11.9 (7.4–16.5)	0.4
**PYY (pg/ml) (WM)**	59.1 (40.4–99.9)	49.2 (32.6–88.4)	
**PYY (pg/ml) (OF)**	67.0 (41.6–98.0)	56.6 (34.9–107.8)	0.1

Data are means (SD) or median (25%–75%) percentile. WM = weight maintaining diet; OF = overfeeding diet; A-ghrelin = active ghrelin; T-ghrelin = total ghrelin; GLP-1 = glucagon like peptide 1; PYY = pancreatic polypeptide Y_3–36._ P values are analyzed by using paired t-test for A-ghrelin and Wilcoxon test for Threlin, Leptin, GLP-1 and PYY between WM and OF diets comparing the hormone difference before and after each diet.

### Comparison of core temperature during weight maintenance and overfeeding diets

**Figure 3:**
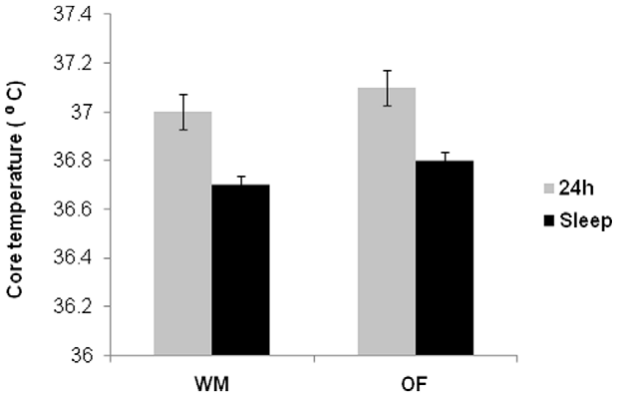
Twenty four hour and sleep core temperature. Mean 24 h (white column) and sleep (gray column) core temperatures were 37.0±0.2 (°C), 36.7±0.2 (°C) respectively on WM, and 37.1±0.2 (°C), 36.8±0.2 (°C) respectively on OF; both mean 24 h core temperature and sleep core temperature did not differ between WM and OF (p = 0.7 and p = 0.5), but mean 24 h core temperature were higher than sleep core temperature both on WM and OF (p = 0.008 and p = 0.008). Temperature data are means ± SD. Paired t-test was used to analyze differences between diets.

Due to core temperature monitor reading issues, only 15 subjects had valid results. Overall mean 24 hour and sleeping core temperature (adjusted for calibration) were not different during WM vs. OF (24 hour: 37.0±0.2°C vs. 37.1±0.2°C, p = 0.7, and sleeping: 36.7±0.2°C vs. 36.8±0.2°C, p = 0.5). As expected, core temperature decreased with sleep: the difference between 24 h hour core temperature and sleep core temperature was 0.3±0.1°C (p = 0.008) during both WM and OF (Figure 3).

### Comparison of non-exercise activity during weight maintenance and overfeeding diets, and correlation of physical activity with weight gain during OF

**Figure 4:**
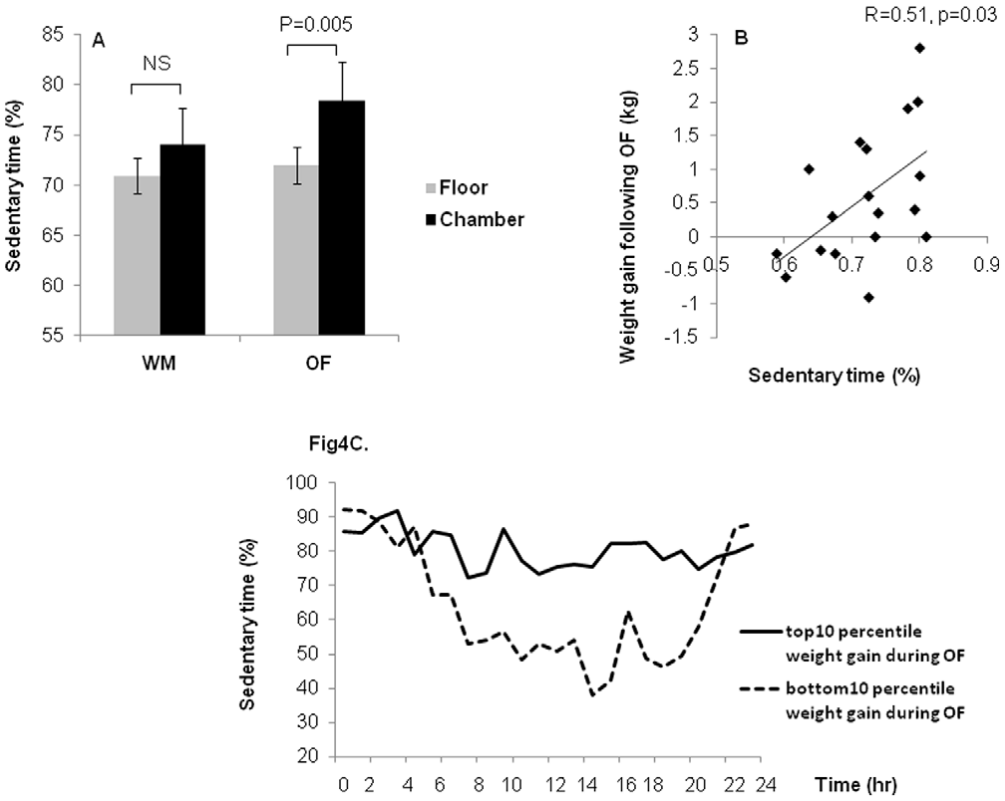
Comparison of sedentary time between weight maintenance diet (WM) and overfeeding diet (OF) and the correlation of sedentary time with age and weight gain during OF. Figure 4A, Sedentary time shown on the inpatient unit and in the chamber on WM (70.9±12.9% and 74.6±10.6%, respectively)and on OF (72.0±7.4% and 78.4±6.6%, respectively). Sedentary time did not differ between WM Vs. OF on the inpatient unit or in the chamber, but increased while in the chamber vs. on the inpatient unit while on OF (p = 0.0005), not on WM. Figure 4B., Sedentary time was positively associated with weight gain during OF (r = 0.51, p = 0.03); Fig. 4C., Sedentary time shown in 24 hours in those with weight gain in top 10 and in bottom 10 percentile during OF. Time shown starting at midnight (0 on x-axis). Data shown as means ± SD. Comparison of sedentary time between diets analyzed using paired t-test; comparison between sedentary time on inpatient unit vs. chamber analyzed using t-test. R values are Pearson correlations.

Nineteen subjects were analyzed on WM diet (1 subject was an outlier and 3 had invalid readings due to technical issues) and20 subjects were analyzed on OF diet (3 had invalid readings due to technical issues). Mean weight change during WM was −0.1±0.7 (kg) and during OF was 0.7±1.0 (kg). The intensity of non-exercise activity as measured at each limb and at the waist was not different between WM and OF (Figure S1). Overall, sedentary time, as calculated from the waist measurement was not different between WM and OF while living on the inpatient unit (70.9±12.9 vs.72.0±7.4%, p = 0.8) or in the metabolic chamber (74.6±10.6 vs. 78.4±6.6%, p = 0.5) (Figure 4A). In 15 individuals, sedentary time on the inpatient unit was averaged over 2 days, in 4 individuals during WM diet and 5 during OF diet who did not go into the chamber sedentary time was averaged over 3 days. Sedentary time was positively associated with weight gain during OF (r = 0.51, p = 0.03) (Figure 4B), but not during WM (r = 0.02, p = 0.6). There was a trend towards an association between initial body weight and weight gain (r = 0.44, p = 0.07). The association between sedentary time and weight gain was still significant after adjustment for diet order (r = 0.49, p = 0.04) and after adjustment in a separate model for age, sex and initial body weight, (r  = 0.49, p = 0.05). Although when age, sex, initial body weight and diet order were all included, the association was attenuated (r = 0.45, p = 0.09). In addition, sedentary time was positively correlated with daily energy balance (r = 0.65, p = 0.02) (calculated as the difference in calories between the overfeeding diet and 24 h EE)) during OF, however, daily energy balance was not correlated with weight gain (r = 0.22, p = 0.5). As noted in figure 4C which showed top versus bottom 10% by weight gain during OF, the increase in sedentary time appears to occur throughout the daytime period.

As the metabolic chamber is a more enclosed space than the research unit which further restricts activity, we investigated the difference in sedentary time while volunteers were living on the regular inpatient unit versus in the metabolic chamber while on each diet. During WM, sedentary time was not different between the inpatient unit and the metabolic chamber (p = 0.6), but during OF sedentary time was significantly higher while subjects were in the metabolic chamber (p = 0.0005) (Figure 4A). Although sedentary time was associated with weight change, we did not find any association between non-exercise activity as measured from waist, wrists and ankles and weight change during OF (p = 0.5, p = 0.8, p = 0.7, respectively). In addition, none of the fasting hormone concentrations before the diet were associated with sedentary time during WM or with sedentary time during OF (data not shown)

### Comparison of 24 h and sleep EE during the weight maintenance and overfeeding diets

Due to respiratory chamber operation issues, only 15 study volunteers have complete metabolic chamber results. 24 h EE and sleep EE significantly increased by 233±168 kcal/day and 173±165 kcal/day (p<0.0001; p = 0.0008) in response to OF compared to WM. In addition, neither 24 h EE nor sleep EE was correlated with mean 24 h core temperature or sleep core temperature (Table S2). Furthermore, there was no association of energy expenditure in the metabolic chamber with mean daily energy intake during subsequent ad libitum feeding (p = 0.8). Spontaneous physical activity (SPA) measured in the chamber was not correlated with weight change during OF (p = 0.6), nor during WM (p = 0.9).

## Discussion

In this study investigating the effects of short term overfeeding, there was no down regulation of food intake to compensate for short term (3 day) overfeeding. Increased weight gain during OF was not associated with a decrease in energy intake. Core body temperature, physical activity and sedentary time were not different between WM and OF. In a secondary analysis we did find an association between sedentary time and weight gain during OF. Despite this association, overall there is minimal compensation for short term positive energy balance during or following overeating.

In our food rich environment overeating is common, and therefore weight stability results only if individuals down regulate caloric intake following periods of overeating or up-regulate physical activity during or following these periods. Negative feedback regulation in food intake has been demonstrated in rats [17]–[20] , but these results have not been reproduced in humans [1], [2], [21], [22]. In men, energy intake was not decreased following a one day period of overfeeding [22]. Following a more prolonged overfeeding period (13 days), Levitsky et al reported that despite significant weight gain and complaints of participants at the end of the overfeeding period about feeling “stuffed", mean daily energy intake decreased but only to the previous baseline levels [2]. Similarly, Jebb et al demonstrated an absence of compensatory adjustments to energy overload in non-obese men [21]. Following a 3 day overfeeding period in our study volunteers still overate when given the opportunity to eat ad libitum from vending machines, and on average the mean caloric intake over the 3 days was not different from that following their WM period. Although both 24 h energy expenditure and sleeping energy expenditure increased significantly by 233±168 kcal and 173±165 kcal during OF compared with WM, the increase was much less than the increase of extra energy taken in by overfeeding (1411 kcal/day). Therefore even a positive energy balance of approximately 1200 kcal/day (1411–233 kcal) was not sufficient to induce a decrease in energy intake. Our data and the previous studies indicate that alteration of total energy intake is not acutely and precisely regulated following periods of overfeeding.

Moreover, we have previously documented that subjects overeat while using our vending machines. We have speculated that this occurs because the volunteers were provided with foods they preferred and an overabundance of food was supplied. In addition, behaviors that inhibit usual food intake by social pressures disappear when eating in this environment. The withdrawal of these social uncover drives to eat. Our method of measuring ad libitum food intake is also highly reproducible. One may speculate that subjects consumed higher fat composition during the ad libitum period in response to the relatively low fat composition on the WM or OF diets. However given that in a previous study, selective reductions in carbohydrate or fat failed to show changes in subsequent food intake, we do not believe that the relatively low fat composition contributed to the overeating [23].

In mice and human studies, exogenous peripheral or central administration of insulin, leptin, PYY, and GLP-1 reduced food intake by acting as a meal ending satiety signal [24]–[27]. Ghrelin is the only hormone known to increase food intake [28]. However these studies were performed with peripheral infusion of hormones thus increasing the circulating hormone concentrations to pharmacologic concentrations [29]. We did not find any correlation between fasting leptin, active ghrelin, total ghrelin and PYY and the changes in these hormones with overfeeding with energy and macronutrient intake. Although previous studies have shown changes in insulin, leptin, GLP-1, PYY, and ghrelin change from fasting to post-fed states [30]–[32], we did not find any difference in these hormones after overfeeding indicating that these hormones are not very sensitive to short term periods of positive energy balance. The current study also agrees with our previous finding that fasting ghrelin concentrations are not affected by short-term overfeeding and do not predict ad libitum food intake in humans [4].

Overall, we did not find any differences in either sedentary time or non-exercise activity (counts/min) during WM and OF. However, in a secondary analysis we did find a positive association between sedentary time and weight gain during OF, which was not observed during WM. This association remained significant after adjustment for diet order, and after separate adjustment for age, sex and initial body weight. However, adjustment for all four terms did attenuate this association although in this analysis this number of terms likely resulted in over-fitting of our model. Our results imply that compensation for positive energy balance which occurs via overeating may in part be regulated through changes in activity (primarily decreases in sedentary time). Murgatroyd el al also did not find an increase in overall physical activity with overfeeding, but did demonstrate that increased activity during overfeeding was essential to avoid a positive energy balance [33]. Similarly, Levine et al demonstrated using more sophisticated monitors that increases in non-exercise activity thermogenesis was associated with less weight gain during overfeeding [6]. We did not observe a difference in total physical activity as assessed by average accelerometry counts per minute. Activity levels while admitted to the clinical research unit were low in all conditions and it does not appear that overfeeding suppressed activity further. It is possible that the activity monitors we used were not sensitive enough to detect reductions in an already low level of physical activity.

It is also possible that overfeeding and weight gain affect sedentary time rather than vice versa. Weight gain has been shown to lead to an increase in sedentary time [34], [35]. In fact, if we assume that sedentary time would reflect time spent at resting energy expenditure, and as energy expenditure (24hEE and sleep EE, a surrogate for resting energy expenditure) increased with overfeeding, it is difficult to account for how the change in sedentary time would account for such a dramatic positive energy balance in some individuals. The positive association between sedentary time and energy balance during OF would seem to support an effect of overfeeding on sedentary behavior. However, energy balance was not associated with weight gain indicating that there are other factors, such as resistance to the increase in sedentary time, which may affect weight. Nevertheless, the direction of causality is not completely clear in our study. Our finding does suggest that how weight gain and sedentary time impact each other is an area that requires further study.

It is interesting that during overfeeding, further confinement (within a metabolic chamber) lead to a significant increase in sedentary time. This indicates overeating combined with spaces which restrict activity may even further promote inactivity. Our data would seem to be consistent with the observed associations between more confining, sedentary activities (e.g. TV watching, computer time) and adiposity [34], [35]. Therefore, it is reasonable to speculate that the combination of more confined work spaces and our food rich environment are even further spurring our current obesity epidemic. In animals, leptin and ghrelin play roles in the regulation of spontaneous physical activity [36], [37]. However, we did not find any association between circulating hormones and activity intensity or sedentary time on either diet which might explain our associations.

It should be noted that overfeeding induced a change in energy expenditure but not in core body temperature. Some obese mice models have lower core body temperatures compared with lean mice [38], [39] implicating a link between temperature and increased adiposity. The role of body temperature in human adiposity is not as clear as it has not been reported to differ between lean and obese individuals [5]. Our core temperature findings indicate that it does not play a key role in the OF associated increase in EE. The increase in TEE between the WM and OF diets was not related to changes in core body temperature or non-exercise activity indicating that other mechanisms such as gastrointestinal processes or changes in sympathetic tone may mediate this increase in 24EE.

We must acknowledge that our vending machine model of ad libitum food intake results in the tendency to overeat [13]. However, this model of energy intake has been shown to be highly reproducible and permits exact recording of calorie intake. In addition, although this study was relatively small, the power of our study was enhanced as our cross over design increased our power to detect differences following each diet.

It is also true that although the diets were given in random order, volunteers were not blinded to the diets, thus it is unclear whether the observed changes in sedentary time were a more willful attempt to prevent weight gain or a less conscious changes in activity. In addition, we acknowledge that our primary outcome for this analysis was ad libitum food intake. Thus, our other findings including those related to sedentary time but also to core temperature, non-exercise activity and fasting hormone concentrations were part of secondary analyses and should be treated with appropriate caution. Also, less variance in weight change in the WM could have masked similar associations. We also acknowledge that we only measured fasting hormones postprandial concentrations of those hormones may have yielded different results.

We demonstrated that in humans, during and following short term overfeeding (3 day) there was no change in physical activity, sedentary time, core body temperature or subsequent ad libitum food intake. In a secondary analysis, we did observe an association between weight gain and sedentary time during overfeeding. Whether such changes in sedentary time are a response to weight change or mediate this change is not clear. Overall, overfeeding results in minimal attempts to restore energy balance. Given our food rich, activity restricted environment, opportunities to decrease sedentary time to compensate for overfeeding might mitigate, at least in part, the problem of increasing adiposity.

## Supporting Information

Table S1 **Spearman correlations for fasting circulating hormone concentrations with ad libitum daily energy and carbohydrate intake.**
(PPTX)

Table S2 **Spearman Correlations between 24 h and sleep energy expenditure and 24 h and sleep core temperature.**
(PPTX)

Figure S1 **Summary of physical activity counts.** Non-exercise physical activity measured from each wrist, ankle and waist. Counts were expressed by the Actical in device-specific arbitrary units (counts per minute). Counts compared by paired t-test. There were no differences non-exercise activity on LA, RA, LW, RW, W between WM Vs. OF (p = 0.7 and p = 0.9 for LW and RW respectively) and (p = 0.9 for LW and RW) and (p = 0.6 for waist). LA = left ankle; RA = right ankle; LW = left wrist; RW = right wrist; W = waist. WM = weight maintaining diet; OF = overfeeding diet. Differences between diets analyzed using paired t-test.
(PPTX)

Checklist S1 **CONSORT Checklist.** CONSORT 2010 checklist of information to include when reporting a randomised trial.
(DOC)

Flowchart S1 **CONSORT 2010 flow diagram.**
(DOCX)

Protocol S1 **Trial Protocol.** OH99-DK-N019.
(DOCX)

## References

[1] Levitsky DA (2005). The non-regulation of food intake in humans: hope for reversing the epidemic of obesity.. Physiol Behav.

[2] Levitsky DA, Obarzanek E, Mrdjenovic G, Strupp BJ (2005). Imprecise control of energy intake: absence of a reduction in food intake following overfeeding in young adults.. Physiol Behav.

[3] Salbe AD, Tschop MH, DelParigi A, Venti CA, Tataranni PA (2004). Negative relationship between fasting plasma ghrelin concentrations and ad libitum food intake.. J Clin Endocrinol Metab.

[4] Votruba SB, Kirchner H, Tschop M, Salbe AD, Krakoff J (2009). Morning ghrelin concentrations are not affected by short-term overfeeding and do not predict ad libitum food intake in humans.. Am J Clin Nutr.

[5] Savastano DM, Gorbach AM, Eden HS, Brady SM, Reynolds JC (2009). Adiposity and human regional body temperature.. Am J Clin Nutr.

[6] Levine JA, Eberhardt NL, Jensen MD (1999). Role of nonexercise activity thermogenesis in resistance to fat gain in humans.. Science.

[7] Westerterp KR (2010). Physical activity, food intake, and body weight regulation: insights from doubly labeled water studies.. Nutr Rev.

[8] Norgan NG, Durnin JV (1980). The effect of 6 weeks of overfeeding on the body weight, body composition, and energy metabolism of young men.. Am J Clin Nutr.

[9] Stubbs RJ, van Wyk MC, Johnstone AM, Harbron CG (1996). Breakfasts high in protein, fat or carbohydrate: effect on within-day appetite and energy balance.. Eur J Clin Nutr.

[10] Pannacciulli N, Salbe AD, Ortega E, Venti CA, Bogardus C (2007). The 24-h carbohydrate oxidation rate in a human respiratory chamber predicts ad libitum food intake.. Am J Clin Nutr.

[11] Rising R, Alger S, Boyce V (1992). Food intake measured by an automated food-selection system: relationship to energy expenditure.. Am J Clin Nutr.

[12] Geiselman PJ, Anderson AM, Dowdy ML, West DB, Redmann SM (1998). Reliability and validity of a macronutrient self-selection paradigm and a food preference questionnaire.. Physiol Behav.

[13] Venti CA, Votruba SB, Franks PW, Krakoff J, Salbe AD (2010). Reproducibility of ad libitum energy intake with the use of a computerized vending machine system.. Am J Clin Nutr.

[14] Crouter SE, Bassett DR (2008). A new 2-regression model for the Actical accelerometer.. Br J Sports Med.

[15] Ravussin E, Lillioja S, Anderson TE, Christin L, Bogardus C (1986). Determinants of 24-hour energy expenditure in man. Methods and results using a respiratory chamber.. J Clin Invest.

[16] Nguyen T, de Jonge L, Smith SR, Bray GA (2003). Chamber for indirect calorimetry with accurate measurement and time discrimination of metabolic plateaus of over 20 min.. Med Biol Eng Comput.

[17] Christy LA, Arvinth S, Saravanakumar M (2009). Engineering sugarcane cultivars with bovine pancreatic trypsin inhibitor (aprotinin) gene for protection against top borer (Scirpophaga excerptalis Walker).. Plant Cell Rep.

[18] Harris RB, Kasser TR, Martin RJ (1986). Dynamics of recovery of body composition after overfeeding, food restriction or starvation of mature female rats.. J Nutr.

[19] Hoebel BG, Teitelbaum P (1966). Weight regulation in normal and hypothalamic hyperphagic rats.. J Comp Physiol Psychol.

[20] Seeley RJ, Matson CA, Chavez M, Woods SC, Dallman MF (1996). Behavioral, endocrine, and hypothalamic responses to involuntary overfeeding.. Am J Physiol.

[21] Jebb SA, Siervo M, Fruhbeck G, Goldberg GR, Murgatroyd PR (2006). Variability of appetite control mechanisms in response to 9 weeks of progressive overfeeding in humans.. Int J Obes (Lond).

[22] Johnstone AM, Stubbs RJ, Harbron CG (1996). Effect of overfeeding macronutrients on day-to-day food intake in man.. Eur J Clin Nutr.

[23] Penesova A, Venti CA, Bunt JC, Bonfiglio SM, Votruba SB (2011). Short-term isocaloric manipulation of carbohydrate intake: effect on subsequent ad libitum energy intake.. Eur J Nutr.

[24] Ahima RS, Kelly J, Elmquist JK, Flier JS (1999). Distinct physiologic and neuronal responses to decreased leptin and mild hyperleptinemia.. Endocrinology.

[25] Batterham RL, Cohen MA, Ellis SM (2003). Inhibition of food intake in obese subjects by peptide YY3-36.. N Engl J Med.

[26] Meeran K, O'Shea D, Edwards CM (1999). Repeated intracerebroventricular administration of glucagon-like peptide-1-(7–36) amide or exendin-(9–39) alters body weight in the rat.. Endocrinology.

[27] Vettor R, Fabris R, Pagano C, Federspil G (2002). Neuroendocrine regulation of eating behavior.. J Endocrinol Invest.

[28] Wren AM, Seal LJ, Cohen MA (2001). Ghrelin enhances appetite and increases food intake in humans.. J Clin Endocrinol Metab.

[29] Geary N (2004). Endocrine controls of eating: CCK, leptin, and ghrelin.. Physiol Behav.

[30] Cummings DE, Overduin J (2007). Gastrointestinal regulation of food intake.. J Clin Invest.

[31] Havel PJ (2001). Peripheral signals conveying metabolic information to the brain: short-term and long-term regulation of food intake and energy homeostasis.. Exp Biol Med (Maywood.).

[32] Karra E, Batterham RL (2010). The role of gut hormones in the regulation of body weight and energy homeostasis.. Mol Cell Endocrinol.

[33] Murgatroyd PR, Goldberg GR, Leahy FE, Gilsenan MB, Prentice AM (1999). Effects of inactivity and diet composition on human energy balance.. Int J Obes Relat Metab Disord.

[34] Hu FB, Li TY, Colditz GA, Willett WC, Manson JE (2003). Television watching and other sedentary behaviors in relation to risk of obesity and type 2 diabetes mellitus in women.. JAMA.

[35] Livingstone MB, Robson PJ, Wallace JM, McKinley MC (2003). How active are we? Levels of routine physical activity in children and adults.. Proc Nutr Soc.

[36] Horvath TL, Diano S, Sotonyi P, Heiman M, Tschop M (2001). Minireview: ghrelin and the regulation of energy balance–a hypothalamic perspective.. Endocrinology.

[37] Ahima RS, Bjorbaek C, Osei S, Flier JS (1999). Regulation of neuronal and glial proteins by leptin: implications for brain development.. Endocrinology.

[38] Klaus S, Munzberg H, Truloff C, Heldmaier G (1998). Physiology of transgenic mice with brown fat ablation: obesity is due to lowered body temperature.. Am J Physiol.

[39] Trayhurn P, James WP (1978). Thermoregulation and non-shivering thermogenesis in the genetically obese (ob/ob) mouse.. Pflugers Arch.

