# Supplementary material for: Measurement of Ad Libitum Food Intake, Physical Activity, and Sedentary Time in Response to Overfeeding
Source: PLoS One. 2012 May 22;7(5):e36225. doi: 10.1371/journal.pone.0036225 (PMC3358301; doi:10.1371/journal.pone.0036225)
Supplement: Table S1 — Spearman correlations for fasting circulating hormone concentrations with ad libitum daily energy and carbohydrate intake. (PPTX) [file pone.0036225.s001.pptx]

## Slide 1
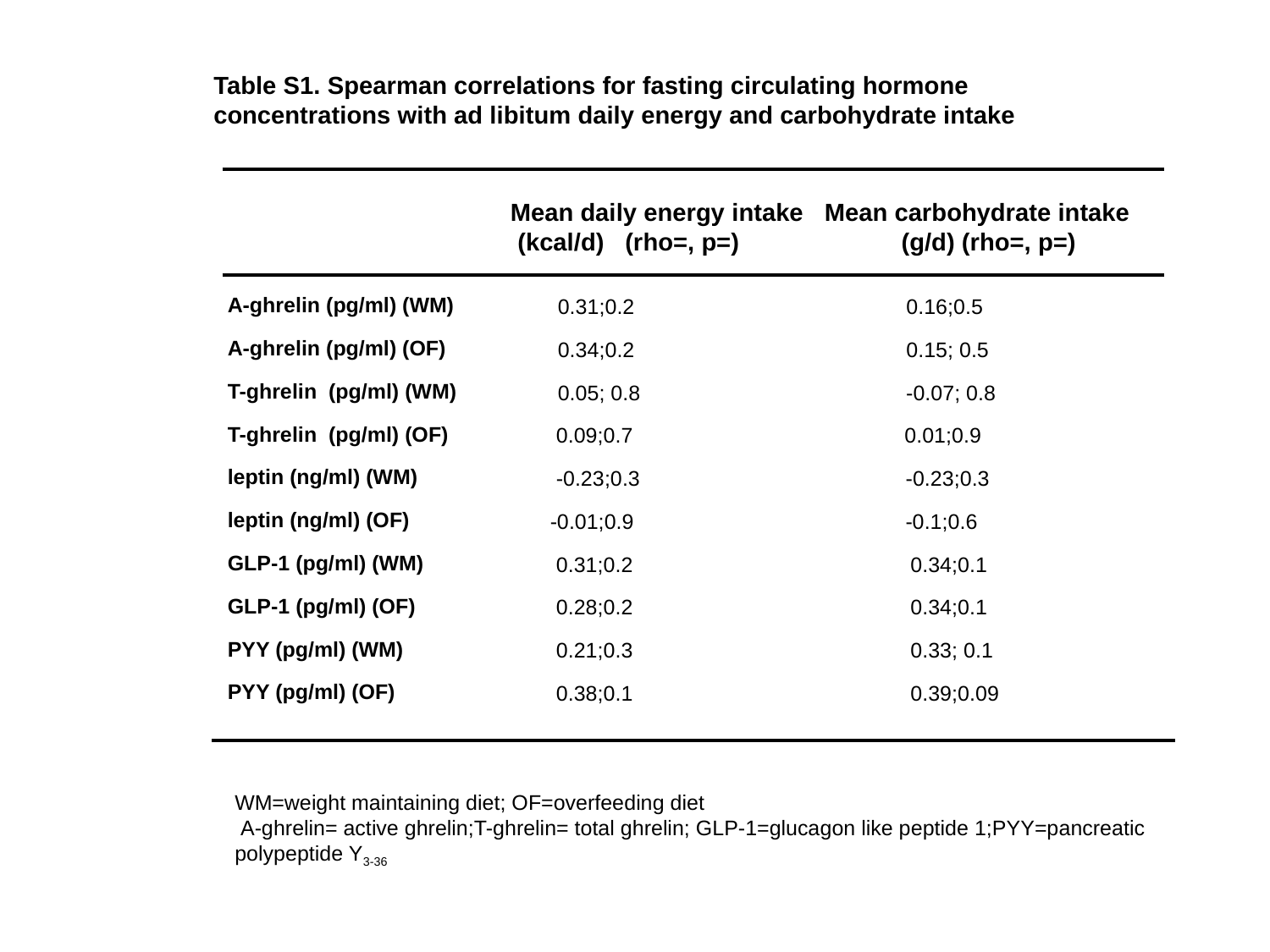

Table S1. Spearman correlations for fasting circulating hormone concentrations with ad libitum daily energy and carbohydrate intake
Mean daily energy intake
 (kcal/d) (rho=, p=)
 Mean carbohydrate intake
 (g/d) (rho=, p=)
 0.31;0.2 0.16;0.5
 0.34;0.2 0.15; 0.5
 0.05; 0.8 -0.07; 0.8
 0.09;0.7 0.01;0.9
 -0.23;0.3 -0.23;0.3
 -0.01;0.9 -0.1;0.6
 0.31;0.2 0.34;0.1
 0.28;0.2 0.34;0.1
 0.21;0.3 0.33; 0.1
 0.38;0.1 0.39;0.09
 A-ghrelin (pg/ml) (WM)
 A-ghrelin (pg/ml) (OF)
 T-ghrelin (pg/ml) (WM)
 T-ghrelin (pg/ml) (OF)
 leptin (ng/ml) (WM)
 leptin (ng/ml) (OF)
 GLP-1 (pg/ml) (WM)
 GLP-1 (pg/ml) (OF)
 PYY (pg/ml) (WM)
 PYY (pg/ml) (OF)
WM=weight maintaining diet; OF=overfeeding diet
 A-ghrelin= active ghrelin;T-ghrelin= total ghrelin; GLP-1=glucagon like peptide 1;PYY=pancreatic polypeptide Y3-36
