# Supplementary material for: Measurement of Ad Libitum Food Intake, Physical Activity, and Sedentary Time in Response to Overfeeding
Source: PLoS One. 2012 May 22;7(5):e36225. doi: 10.1371/journal.pone.0036225 (PMC3358301; doi:10.1371/journal.pone.0036225)
Supplement: Table S2 — Spearman Correlations between 24 h and sleep energy expenditure and 24 h and sleep core temperature. (PPTX) [file pone.0036225.s002.pptx]

## Slide 1
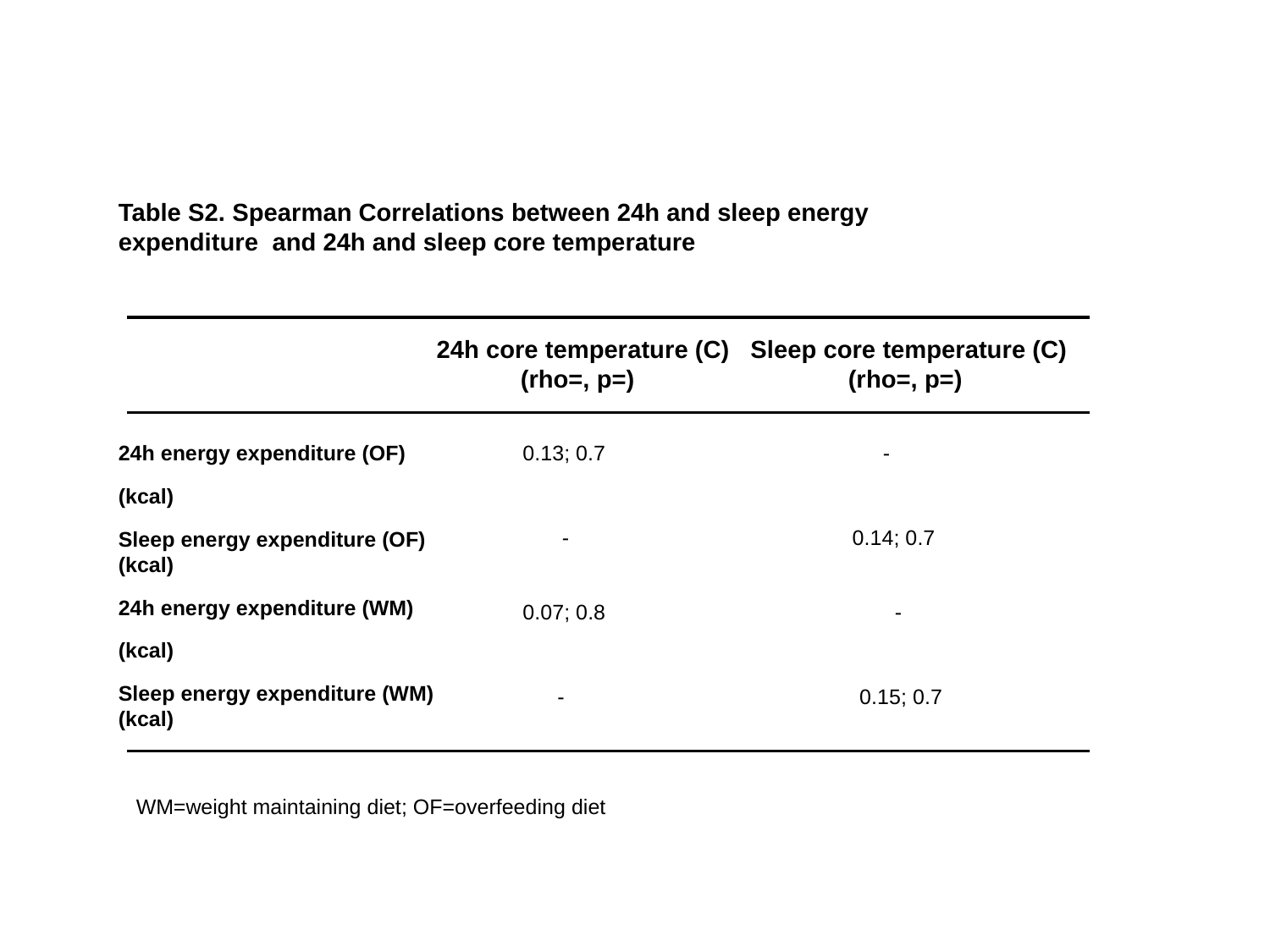

Table S2. Spearman Correlations between 24h and sleep energy expenditure and 24h and sleep core temperature
24h core temperature (C)
 (rho=, p=)
 Sleep core temperature (C)
 (rho=, p=)
24h energy expenditure (OF)
(kcal)
Sleep energy expenditure (OF) (kcal)
24h energy expenditure (WM)
(kcal)
Sleep energy expenditure (WM) (kcal)
0.13; 0.7 -
 - 0.14; 0.7
0.07; 0.8 -
 - 0.15; 0.7
WM=weight maintaining diet; OF=overfeeding diet
