# Supplementary figures and images for: Measurement of Ad Libitum Food Intake, Physical Activity, and Sedentary Time in Response to Overfeeding
Source: PLoS One. 2012 May 22;7(5):e36225. doi: 10.1371/journal.pone.0036225 (PMC3358301; doi:10.1371/journal.pone.0036225)

## Slide 1
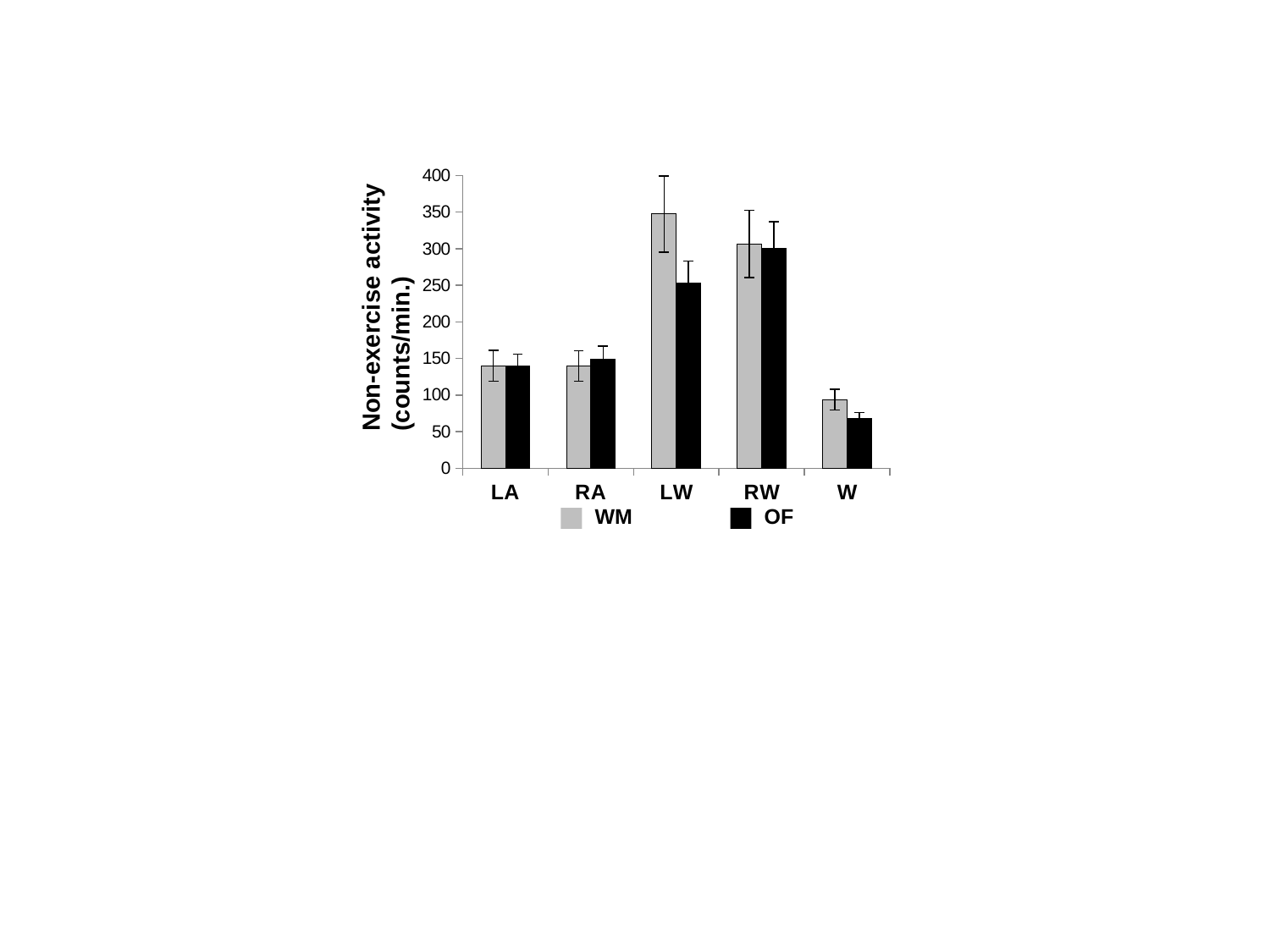

### Chart
| Category | | |
|---|---|---|
| LA | 140.05478600150002 | 139.25 |
| RA | 139.69394852500002 | 149.0 |
| LW | 347.34374900999995 | 253.0 |
| RW | 306.376264675 | 301.0 |
| W | 93.94745218777783 | 68.0 |Non-exercise activity (counts/min.)
WM
OF

Supplement: Figure S1 — Summary of physical activity counts. Non-exercise physical activity measured from each wrist, ankle and waist. Counts were expressed by the Actical in device-specific arbitrary units (counts per minute). Counts compared by paired t-test. There were no differences non-exercise activity on LA, RA, LW, RW, W between WM Vs. OF (p = 0.7 and p = 0.9 for LW and RW respectively) and (p = 0.9 for LW and RW) and (p = 0.6 for waist). LA = left ankle; RA = right ankle; LW = left wrist; RW = right wrist; W = waist. WM = weight maintaining diet; OF = overfeeding diet. Differences between diets analyzed using paired t-test. (PPTX) [file pone.0036225.s003.pptx]
