# Supplementary material for: Measurement of Ad Libitum Food Intake, Physical Activity, and Sedentary Time in Response to Overfeeding
Source: PLoS One. 2012 May 22;7(5):e36225. doi: 10.1371/journal.pone.0036225 (PMC3358301; doi:10.1371/journal.pone.0036225)
Supplement: Flowchart S1 — CONSORT 2010 flow diagram. (DOCX) [file pone.0036225.s005.docx]

**CONSORT 2010 Flow Diagram**

Analysed (n=19 ), non exercise activity evaluated as sedentary time
♦ Excluded from analysis (give reasons) (n=4 ), invalid readings from actical

Analysed (n= 15 ) for core temperature
 Excluded from analysis (give reasons) (n= 8), invalid readings or failure in readings

Analysed (n= 15 ) for chamber
♦ Excluded from analysis (give reasons) (n= 8), invalid readings or failure in measuring

## Follow-Up (N/A)

Analysed (n= 21 ) for ad libitum food intake
♦ Excluded from analysis (give reasons) (n= 2 ), invalid food intake data

## Analysis

Lost to follow-up (give reasons) (n= )

Discontinued intervention (give reasons) (n= )

Lost to follow-up (give reasons) (n= )

Discontinued intervention (give reasons) (n= )

## Enrollment

Allocated to Overfeeding diet (n= 12 )

♦ Received overfeeding diet (n= 12 )

♦ Did not receive allocated intervention (give reasons) (n= 0 )

## Allocation (N/A)

Allocated to Weight maintenance diet (n=11 )

♦ Received weight maintenance diet (n=11 )

♦ Did not receive allocated intervention (give reasons) (n= 11 )

Randomized (n=23)

Excluded (n= 8 )

♦  Not meeting inclusion criteria (n=8 )

♦  Declined to participate (n= 0 )

♦  Other reasons (n= 0 )

Assessed for eligibility (n=31 )
