# Supplementary material for: Measurement of Ad Libitum Food Intake, Physical Activity, and Sedentary Time in Response to Overfeeding
Source: PLoS One. 2012 May 22;7(5):e36225. doi: 10.1371/journal.pone.0036225 (PMC3358301; doi:10.1371/journal.pone.0036225)
Supplement: Protocol S1 — Trial Protocol. OH99-DK-N019. (DOCX) [file pone.0036225.s006.docx]

**The Food Intake Phenotype: Assessing Eating Behavior and**

**Food Preferences as Risk Factors for Obesity**

Principal Investigator / Accountable Investigator:

Clifton Bogardus, MD

PECRB, NIDDK

445 N 5^th^ St, Ste 2A-20

Phoenix, AZ 85004

Tel: 602-440-6570

[cbogardu@mail.nih.gov](mailto:cbogardu@mail.nih.gov)

Type: Natural History – Sample/Data Collection/Analysis: Recruiting

Lead Associate Investigator:

Susanne B Votruba, PhD ODCRS, PECRB, NIDDK 602-200-5336

[votrubas@mail.nih.gov](mailto:votrubas@mail.nih.gov)

Associate Investigators:

Joy C Bunt, MD, PhD ODCRS, PECRB, NIDDK 602-200-5312

[jbunt@mail.nih.gov](mailto:jbunt@mail.nih.gov)

About the Protocol:

Uses Ionizing Radiation? Yes

Requires IND/IDE? No

Sponsor: None

Uses “Durable Power of Attorney”? No

Study Location: Clinical Research Unit, NIDDK-Phoenix

Multi-Institutional Project? No

Expedited Review Request? No

**PRECIS**

The prevalence of obesity in the United States has reached alarming proportions with 33% of adults over the age of 20 being overweight. In some populations, obesity is even more prevalent. Although there have been a number of advances in our understanding of the genetics of obesity, the environmental influences on the genetic expression of obesity requires further investigation.

In an effort to understand some of the influences on the high prevalence of obesity, the present study was designed to investigate what drives how much people eat. More specifically, we will try to understand what drives food intake utilizing 1) questionnaires that assess eating behavior, 2) measurements in blood, urine or fat tissue, and 3) genotypic associations to investigate the various factors that control what and how much people eat. As food intake influences appetite-regulating hormones and vice versa, we also plan to assess the effect of short-term overfeeding on ad libitum intake and the appetite-stimulating hormone, ghrelin. Also, as the body’s metabolism of carbohydrates may influence food intake, we propose to look at the effects of isocaloric high fat and high carbohydrate diets on short-term ad libitum intake. We will make these evaluations by assessing eating behavior, food preferences including usual fat intake and preferences for high fat foods, body image perceptions, and energy expenditure. It is hoped that the data gathered from this study will elucidate some of the risk factors for the development of obesity.

**INTRODUCTION**

The prevalence of overweight (defined as body mass index [BMI] >25) in the United States was recently reported to be approximately 33% in adults 20 years of age and older, (1) an increase of 8% between the NHANES surveys conducted between 1976-1980 and 1988-1991. Because obesity is a multi-factorial disorder having both environmental and genetic determinants (2), understanding the etiology of this disorder remains among the most difficult problems facing the nutrition community. While some of the determinants of weight gain have been identified (3), much remains to be understood about the factors influencing eating behavior (4), especially the preference for high fat foods, and whether this preference is under genetic control.

One of the published reports generated from this study indicated a negative relationship between fasting plasma total ghrelin concentrations and *ad libitum* food intake as recorded by the computerized vending machines (5). This is particularly interesting in light of other studies suggesting that ghrelin stimulates food intake over the short term. It has also been reported, however, that obese individuals have blunted circulating ghrelin levels, and that obese Caucasians have higher ghrelin levels than obese Pima Indians (6). Furthermore, Ravussin et al. (7) have shown in twin studies that ghrelin concentrations tended to decrease or increase concurrently with overfeeding or negative energy balance, respectively. These data indicate that total ghrelin concentrations reflect chronic energy balance, while still having acute effects on food intake. As total ghrelin concentrations indicate previous chronic energy balance, it is possible that our previous surprising findings of a negative association between total ghrelin concentrations and food intake reflect previous positive energy balance (e.g., overeating) prior to admission to the clinical research unit. This would decrease total ghrelin concentrations in volunteers consuming greater calories and account for our associations.

Energy intake is also known to be an important mediator of thyroid hormone action. T3 was increased and rT3 decreased in both long term (8) and short term (9) over-feeding, while T4 and TSH remained unchanged. Additionally, thyroid hormone and catecholamines, including epinephrine and norepinephrine, are some of the main hormones that regulate resting metabolic rate. Low T3 and sympathetic nerve activity are correlated with lower energy expenditure, and both predict weight gain over time (10;11). Yet, it is still unclear if increased energy expenditure after overfeeding is from the adaptation of thyroid hormones and/or sympathetic nerve activity, which is measurable via plasma catecholamines.

Eating behavior is said to bridge the gap between the nutritional environment and the biological mechanisms of weight control (12). The quantity and quality of food consumed, the frequency of meal consumption, and the factors motivating one to eat are important aspects of food intake regulation. Using twin studies, de Castro (13) has shown that daily intake, including macronutrient intake and meal patterns, is genetically influenced. He found that heredity accounts for between 50-64% of the variance in carbohydrate, fat, protein, alcohol and water intake, 44% of the variance in meal frequency, and 76% of intake expressed in grams. These effects were independent of body size (14) as well as environmental factors. Taste perceptions also appear to be under genetic control. A recent review by Tepper (15) suggests that PROP-tasting, the ability to perceive as bitter the compound 6-n-propylthiouracil, is under genetic control and this ability confers sensitivity to specific taste perceptions and food acceptance.

Many studies have shown that dietary fat intake is a major determinant of body fat (16-19) and fat intake is significantly associated with an increase in body mass index (20). Cooling and Blundell (21) found that appetite control in habitual high-fat consumers is regulated differently than in habitual high-carbohydrate consumers: high-fat consumers appear to eat a constant weight of food whereas high-carbohydrate consumers appear to eat a constant level of energy. There is considerable evidence showing that fat intake has actually been decreasing as the prevalence of obesity has been rising (22-24), suggesting that there are inter-individual differences in fat metabolism that may offer protection from or susceptibility to obesity; it is likely that these differences are under genetic control (18) as well.

Despite this, recent evidence suggests that carbohydrate oxidation and balance as measured by indirect calorimetry during a period of weight maintenance may play pivotal roles in food intake (25). This finding is particularly interesting in light of the report from Eckel et al. (26) showing that a positive carbohydrate balance during a transient period of relative inactivity is associated with weight gain at four years. Their data suggests that those individuals with a greater capacity to expand glycogen stores during times of carbohydrate overfeeding may be better able to regulate their food intake and protect against weight and fat gain over time. These findings are consistent with the Flatt hypothesis (27) that regulation of carbohydrate stores may be what ultimately drives food intake.

We have recently shown that in vitro adipocyte lipolysis is inversely related to weight gain over ~9 years (ADA 2009 annual meeting abstract, #1363-P). This suggests that the physiology of isolated adipocytes may provide valuable information about a person’s ability or propensity to gain weight over time. Therefore, the collection of fat tissue and measurement of adipocyte lipolysis may be useful in developing a marker of weight gain. Furthermore, we postulate that adipocyte lipolysis may be related to ad libitum food intake as a driver of the weight gain.

Dietary self-report has been used intensively for the measurement of food intake, but there are numerous shortcomings, particularly in regards to long-term use. Both food frequency questionnaires (28-30) and self-reported diet diaries (31;32) tend to be inaccurate; people tend to miscalculate and underreport their daily intake. Using cameras to assess food intake appears to be comparable to diet records (33;34) but even when multimedia diet records that include cameras are used, it appears that people still underreport their food intake (35).

The primary hypothesis of this proposal is that food intake, and perhaps more specifically fat intake, is under genetic control and can be identified as such. This genetic control may be manifest via behavioral or physiologic measurements that control food intake, and thus these data need to be collected at the time of the food intake measurement as well. In addition, this model can be used to validate outpatient methods to collect food intake data.

In addition, we have two other arms of this protocol to 1) address the effect of overfeeding on plasma ghrelin concentrations and spontaneous physical activity and their relations to *ad libitum* food intake and 2) to determine the effects of the macronutrient composition of the diet on *ad libitum* food intake. We will use questionnaires, taste testing, food preference studies, spontaneous physical activity monitors, measurement of adipose tissue lipolysis, and measurements of core body temperature to establish a ‘food intake phenotype’ in non-diabetic volunteers from a range of ethnic groups. We hope to be able to show a relationship, for example, between a preference for high fat foods and a genetic polymorphism or perhaps between specific eating behaviors and a polymorphism.

Volunteers may be invited for follow-up studies at one month, 6 months, and 12 months to assess the reproducibility of these measurements as well as the prospective and longitudinal associations. Additionally, based on the previous results (5), for the overfeeding arm of the study, we hypothesize that with a short period of acute overfeeding, ghrelin will suppress to a lesser degree in individuals who subsequently have greater food intake. Therefore, we hypothesize, that while we will still find a negative association between ghrelin and food intake, individuals with greater food intake will also have lesser ghrelin suppression after overfeeding. The hypothesis for the study arm manipulating dietary macronutrient content is that a short-term (3-day) high carbohydrate intake will increase carbohydrate balance and result in lower *ad libitum* food intake compared to a short-term (3-day) high fat diet in the same person. We also propose to follow those study volunteers who participate in these arms of the study longitudinally (at 6 months and 1 year) to assess weight gain over time. The return visits would require a 1-day visit at which we would measure weight, body composition by DEXA and fasting blood tests.

**PROTOCOL**

**Subjects and Recruitment**. Non-diabetic volunteers aged 18-65 years are eligible to participate in this study.

After equilibration to the metabolic unit, an oral glucose tolerance test is one of the first procedures performed. If a subject previously believed to be non-diabetic tests positive for diabetes during this admission, he/she will be provided with diabetes and nutrition education and referred to the appropriate services for follow-up care.

A number of demographic and environmental factors are known to influence food preferences including age, marital status, household composition, finances available for food purchasing, access to shopping facilities, identity of the food provider, and employment status, among others. Many of these factors are considered as part of the patient admission record and where appropriate, will be considered as covariates in the creation of the food intake phenotype. For example, a shared living environment has been shown to affect food intake patterns (36) as well as food preferences (37). Moreover, we will attempt to enroll only one subject per family at any given time so that sharing an environment during the study cannot be said to influence the study outcome.

Inclusion Criteria:

1. Healthy, as determined by medical history, physical examination, and laboratory tests.

Exclusion Criteria:

1. Hypo- or hyper-thyroid

2. Blood pressure greater than 160/95

3. Cardiovascular disease

4. Gallbladder disease

5. Alcohol and/or current use of drugs (more than 2 drinks per day and regular use of drugs such as amphetamines, cocaine, heroin, or marijuana)

6. Psychiatric conditions or behavior that would be incompatible with safe and successful participation in this study, including claustrophobia and eating disorders such as anorexia or bulimia nervosa

8. Use of medications affecting metabolism and appetite

9. Pregnancy

10. Smoking

All subjects will be fully informed of the aim, nature, and risks of the study prior to giving written informed consent. Subjects will also be specifically advised that the results of testing for drug usage will be filed in the medical record and would, therefore, be available as part of this record.

Subjects may be excluded or withdrawn from the study if they have any conditions not specifically mentioned above that may interfere with the collection of the food intake. This includes such issues as not following study and unit policies and procedures, diagnosis of contraindications following admission, and development of illness/infection unrelated to the study. For example, volunteers who do not comply with the vending machine protocol (i.e., share food, do not record what they eat, eat outside of room, etc) may be withdrawn from the study.

**Experimental Design:**

In this protocol, we will investigate eating behavior, caloric intake and food preferences. We will utilize questionnaires and methods of assessing eating behavior, measurements in blood, urine, and fat as well as measurements of substrate oxidation to elucidate physiologic mechanisms that underlie caloric intake as well as investigate genetic polymorphisms that may be related to calorie intake or to behaviors or factors that are associated with caloric intake. Volunteers will also be invited to wear a swallowing collar and band-aid chewing sensors, to validate these in the measurement of food intake. In the two study arms, we will investigate eating behavior and food preferences in response to overfeeding or to changes in the macronutrient content of the diet. Most specifically, we will utilize several questionnaires and methods of assessing eating behavior and the preference for high fat foods to create a ‘food intake phenotype’. We will also draw fasting total and active ghrelin concentrations prior to and following a 3-day period of overfeeding by 50% of weight maintenance caloric needs. This will allow us to confirm that overfeeding suppresses fasting ghrelin concentrations. This will be followed by 3 days of *ad libitum* food intake using the computerized vending machine system. In the macronutrient content of the diet study arm, we will assess the effects of a 3-day period of both (in random order) an isocaloric high fat (50% fat, 30% carbohydrate, 20% protein) and an isocaloric high carbohydrate (60% carbohydrate, 20% fat, and 20% protein) diet on subsequent food intake.

To achieve these goals, we will measure the following:

1. Height and weight

2. Body composition by dual energy X-ray absorptiometry (DXA)

3. Oral glucose tolerance

4. Eating behavior using the Gormally binge eating questionnaire and the Stunkard 3-factor eating questionnaire

5. Food preferences using the Drewnowski taste test, the response to PROP tasting (original study only), and the food preferences questionnaire

6. Fat Intake using the Block 2005 food questionnaire to assess usual nutrient intakes and the vending machines (combined with the pretest foods listing) to assess actual intakes

7. Body image perception using the Stunkard body image questionnaire

8. Sedentary energy expenditure and 24-hour respiratory quotient using the respiratory chamber. This will be repeated 2 times during the overfeeding study arm and 3 times during the changing macronutrients study arm

9. Fasting blood samples for assessment of orexigenic/anorexic hormones

1. Free-living energy expenditure using the doubly-labeled water method (in original study arm only)
2. The Questionnaire on Eating and Weight Patterns ‑ Revised (QEWP‑R) (38): a self-report instrument used for diagnosis of binge eating disorder (BED)
3. Night Eating Diagnostic Scale (NEDS) (39): developed to diagnose night eating syndrome
4. Emotional Appetite Questionnaire (EMAQ): to examine eating response to both positive and negative emotions and situations (40)
5. Perceived Stress Scale (PSS) (41): to assess both the number of stressful events and their impact from daily activities over 7 consecutive days
6. The Inventory for Depressive Symptomatology (IDS –SR) (42): a measure of depressive signs and symptoms, applicable to atypical depression which has been associated with overeating
7. Physical Anhedonia Scale (43): a 61-item scale to assess sensitivity to reward (STR). It is designed to reflect the degree to which individuals take pleasure from, and are motivated to engage in, rewarding.
8. MacArthur Scale of Subjective Social Status (44): contains 2 ladders measuring subjective socio-economic status. Studies have indicated that ladder rankings are more powerful determinants of health-related outcomes than traditional measures of SES (45).
9. U.S. Adult Food Security Survey Module: Six-Item Short Form (46;47): a self-report 6-item module for the assessment of food insecurity over the past 12-months. It classifies households into 3 categories of food security status according to the U.S. food security scale (46) and NHANES shows significant association between food insecurity and obesity.
10. Psychological performance tests for assessment of decision-making ability (Iowa Gambling Task) (48;49), selective attention (Stroop Color Word Test) (50), cognitive executive functioning (Wisconsin Card Sorting Test) (51), and inhibitory control (Go/No Go Task) (52).
11. Core temperature changes with overfeeding using the CorTemp™ Core Body Temperature Monitoring System (HQInc, Palmetto, FL) – this will be done in the overfeeding arm
12. Spontaneous physical activity during overfeeding using both the Acti-graph and Acti-cal systems – this will be done in the overfeeding arm
13. 24-hour food recall on the day after completing the first vending period. This is to assess the accuracy of subjects’ reporting of food intake by comparing their recall to a known quantity of food intake while on the *ad libitum* feeding
14. Fat biopsies of the subcutaneous abdominal and femoral depots for determination of adipocyte size, adipocyte lipolysis, and other measures of fat metabolism
15. The use of non-obtrusive, wearable sensors to objectively estimate food intake occurrence and food intake mass for one day while subjects are using the computerized vending machine system
16. Dietary fat oxidation while in the respiratory chamber using the stable isotope tracer, ^2^H-palmitate (d_31_-palmitic acid)
17. Vending machine exit questionnaire designed to investigate motivation for eating while using the vending machine

**Experimental protocol for original study:**

Day 1 - Admission, medical history and physical examination

-Urine collections for complete urinalysis, urinary HCG (for pregnancy status in females), and drug testing (for marijuana, cocaine and amphetamine metabolites)

-Anthropometric assessments including height, weight, waist and thigh circumferences

-12-lead electrocardiogram (ECG)

-Blood drawing for complete blood count, platelet count, prothrombin time, partial thromboplastin time, electrolytes, plasma urea nitrogen, creatinine, calcium, total protein, albumin, SGOT, gamma-GT, alkaline phosphatase, bilirubin, thyroid stimulating hormone (TSH), lymphocyte transformation to verify previous genotyping and replenish DNA stores

-Begin weight maintenance diet with calories distributed as 50% from carbohydrate, 30% from fat, and 20% from protein

-Food preferences questionnaire

Day 2 -PROP taste test

-Body composition by dual-energy X-ray absorptiometry (DXA)

-Block 2005 food questionnaire

-Psychological questionnaires and computerized performance tests

Day 3-Taste test of sweet and creamy solutions

Day 4 -Oral glucose tolerance test

Day 5 -Needle biopsy of abdominal and femoral subcutaneous adipose tissue

Adipose tissue will be removed by needle biopsy from the abdominal area, usually at McBurney’s point or at the equivalent site on the left side, and from the femoral area, at the anterior-lateral aspect of the thigh, one- to two-thirds of the distance from the superior iliac spine to the patella. After thorough washing and sterilization, local anesthesia of the cutaneous and subcutaneous tissue will be established using 1% lidocaine. The needle biopsy is then performed using a 12 ml plastic syringe and 14-gauge blunt, closed end needle, with an opening on the side. The needle will be repeatedly moved back and forth in the subcutaneous adipose tissue, while maintaining suction on the syringe, until collection of a sufficient amount of sample for study. The adipose tissue is then rapidly exposed to collagenase to obtain isolated cells that can be counted and measured.

Day 6 -Respiratory chamber for assessment of 24-hour sedentary energy expenditure and respiratory quotient

-Urine sample prior to breakfast for baseline deuterium analysis (^2^H)

-Prior to entering respiratory chamber – breakfast, which contains a ^2^H-palmitate tracer to measure meal fat oxidation. Tracer (^2^H) is presented in a liquid meal replacement (Ensure™) as part of the regular breakfast

-Dietary fat oxidation will be assessed by tracer excretion in 24h urine sample collected while in chamber

Days 7-9 -Vending machine study with daily blood drawing for assessment of orexigenic/anorexic hormone levels

Day 7 – Attach chew (strain) sensor and microphone to measure swallowing

-Calibration meal prior to allowing volunteer to eat from the vending machine

-Following calibration, volunteers start eating from vending machine

Day 8 – remove chew and swallow sensors

Day 10 - Blood drawing for assessment of orexigenic/anorexic hormone levels

- Vending machine exit questionnaire

-Discharge

**Experimental protocol for ghrelin suppression and food intake substudy:**

**Day 1** -Admission, medical history and physical examination

-Urine collections for complete urinalysis, urinary HCG (for pregnancy status in females), and drug testing (for marijuana, cocaine and amphetamine metabolites)

-Anthropometric assessments including height, weight, waist and thigh circumferences

-12-lead electrocardiogram (ECG)

-Blood drawing for complete blood count, platelet count, prothrombin time, partial thromboplastin time, electrolytes, plasma urea nitrogen, creatinine, calcium, total protein, albumin, SGOT, gamma-GT, alkaline phosphatase, bilirubin, thyroid stimulating hormone (TSH)

-Begin weight maintenance diet with calories distributed as 50% from carbohydrate, 30% from fat, and 20% from protein

-Food preferences questionnaire

**Day 2** -Body composition by dual-energy X-ray absorptiometry (DXA)

-Block 2005 food questionnaire

**Day 3** -Psychological questionnaires and computerized performance tests

**Day 4** -Oral glucose tolerance test

**Days 5-6** -Begin either overfeeding diet at 150% of weight maintenance energy intake with calories distributed as 50% from carbohydrate, 30% from fat, and 20% from protein or weight maintenance diet (floor diet)

-Fasting blood draw for total and active ghrelin and hormones, including thyroid hormones (TSH, FT3, FT4, rT3) and catecholamines (norepinephrine and epinephrine) before starting diet

-Begin wearing Actiheart and Actical (wear for 3 day diet)

**Day 7** -Respiratory chamber while on study diet (either overfeeding at 150% of chamber weight maintenance diet or standard chamber diet) for assessment of 24-hour sedentary energy expenditure and respiratory quotient

-Swallow CorTemp™ sensor and begin wearing temperature monitor (prior to chamber)

-Remove Actiheart and Actical when exiting chamber

**Days 8-10** -Vending machine study

-Fasting blood draw for total and active ghrelin and hormones, including thyroid hormones (TSH, FT3, FT4, rT3) and catecholamines (norepinephrine and epinephrine) before starting diet

-Daily blood draws for total ghrelin and orexigenic/anorexigenic hormones

**Day 11-13** -Begin weight maintenance diet with calories distributed as 50% from carbohydrate, 30% from fat, and 20% from protein for “wash-out” period

- 24-hour food recall (day 11)

**Day 14-15** -Begin either overfeeding diet at 150% of weight maintenance energy intake with calories distributed as 50% from carbohydrate, 30% from fat, and 20% from protein or regular weight maintenance diet. Diet type will depend on which diet was given days 5-7.

-Fasting blood draw for total and active ghrelin and hormones, including thyroid hormones (TSH, FT3, FT4, rT3) and catecholamines (norepinephrine and epinephrine) before starting diet

-Begin wearing Actiheart and Actical (wear for 3 day diet)

**Day 16** -Respiratory chamber while on study diet (either overfeeding at 150% of chamber weight maintenance diet or standard chamber diet) for assessment of 24-hour sedentary energy expenditure and respiratory quotient

-Swallow CorTemp™ sensor and begin wearing temperature monitor (prior to chamber)

-Remove Actiheart and Actical when exiting chamber

**Days 17-19** -Vending machine study

-Fasting blood draw for total and active ghrelin and hormones, including thyroid hormones (TSH, FT3, FT4, rT3) and catecholamines (norepinephrine and epinephrine) before starting diet

-Daily blood draws for total ghrelin and orexigenic/anorexigenic hormones

**Day 20** - Vending machine exit questionnaire

-Discharge

**Experimental protocol for the effect of macronutrient content on food intake:**

Day 1 -Admission, medical history and physical examination

-Urine collections for complete urinalysis, urinary HCG (for pregnancy status in females), and drug testing (for marijuana, cocaine and amphetamine metabolites)

-Anthropometric assessments including height, weight, waist and thigh circumferences

-12-lead electrocardiogram (ECG)

-Blood drawing for complete blood count, platelet count, prothrombin time, partial thromboplastin time, electrolytes, plasma urea nitrogen, creatinine, calcium, total protein, albumin, SGOT, gamma-GT, alkaline phosphatase, bilirubin, thyroid stimulating hormone (TSH), lymphocyte transformation to verify previous genotyping and replenish DNA stores

-Begin weight maintenance diet with calories distributed as 50% from carbohydrate, 30% from fat, and 20% from protein

-Food preferences questionnaire

Day 2 --Body composition by dual-energy X-ray absorptiometry (DXA)

-Block 2005 food questionnaire

Day 3 - Psychological questionnaires and computerized performance tests

Day 4 -Oral glucose tolerance test

Day 5 -Respiratory chamber for assessment of 24-hour sedentary energy expenditure

and respiratory quotient

-Blood draw for total and active ghrelin

Days 6-7-Begin isocaloric diets with changes in macronutrient composition. Subjects

will be randomized to receive either the high carbohydrate (HC: 60%

carbohydrate, 20% protein, 20% fat) or high fat (HF: 50% fat, 30% carbohydrate,

20% protein) diet at their weight maintenance energy intake

Day 8 -Respiratory chamber while receiving their chamber weight maintenance energy

intake (same diet as when on floor – HC or HF) for assessment of 24-hour

sedentary energy expenditure and respiratory quotient

Days 9-11-Vending machine study with daily blood drawing for assessment of

orexigenic/anorexic hormone levels

Day 12-14-Begin weight maintenance diet with calories distributed as 50% from

carbohydrate, 30% from fat, and 20% from protein

-Blood drawing for assessment of orexigenic/anorexic hormone levels

- 24-hour food recall (day 12)

Day 15-16 -Begin isocaloric diet with changes in macronutrient composition. Subjects

will receive the HC or HF diet at their weight maintenance energy intake. Diet

type will depend on which diet was given during days 6-8

Day 17-Respiratory chamber while receiving their chamber weight maintenance energy

intake (same diet as when on floor – HC or HF) for assessment of 24-hour

sedentary energy expenditure and respiratory quotient

Day 18-20-Vending machine study with daily blood drawing for assessment of

orexigenic/anorexic hormone levels

Day 21- Vending machine exit questionnaire

-Discharge

**Follow-up visit at 6 and 12 months for ghrelin suppression and food intake and effect of macronutrient content on food intake:**

Day 1: -Fasting blood tests (urine pregnancy test for women)

-Measurement of weight and height

-DXA

**TESTS AND ANALYSES**

**1. Determination of fat preferences and fat intake using the food preferences questionnaire (FPQ) and vending machines to test the macronutrient self-selection paradigm (MSSP).** These procedures are based on a model developed by Geiselman et al. (53) with the express purpose of accurately assessing fat preferences and actual fat intake by varying the fat content of foods systematically with other macronutrients in a model termed the “Macronutrient Self-Selection Paradigm.”

**a. Food preferences questionnaire (day 1).** The food preferences questionnaire (FPQ) consists of a list of food items, each of which falls into one of the following 6 cells:

high fat (>45%)/high sugar (>30%)

low fat (<20%)/high sugar (>30%)

high fat (>45%)/high complex carbohydrate(>30%)

low fat (<20%)/high complex carbohydrate (>30%)

high fat (>45%)/low carbohydrate/high protein (>13%)

low fat (<20%)/low carbohydrate/high protein (>13%)

Many of the foods on the list are from among the top 10 sources of dietary fat in the U.S. including items such as hot dogs, ham and other luncheon meats, doughnuts, cookies, cakes, candies, bread products, muffins, eggs, and cheeses. The listing provided by Dr. Geiselman has been modified to reflect patterns of intake common to Pima Indians and individuals living in the Southwest.

In completing the self-administered FPQ, individuals are asked to rate each food hedonically using a 9-point Likert scale with the following anchors: 1 = dislike extremely; 5 = neutral; 9 = like extremely. In addition, they are asked to indicate how often they eat an item and how often they would prefer to eat an item given a lack of financial and other constraints. Of these items, the foods that are to be made available to the subject while using the vending machines will be those foods which the subject has given an intermediate hedonic rating. No item will be presented to a subject that s/he has ranked below “5."

**b. The vending machine study (days 7-9).** The vending machine study will allow the subject to self-select all of his/her food for 3 (or 7) days from 40 items to be provided in our vending machines. The machines are computer operated, require the use of a security code to access the shelves, and provide information as to the time of day the shelves are accessed. The shelves may be accessed only twice: once to retrieve an item and again to return the leftover remains and wrappings.

Foods provided to the subject will be from the FPQ as indicated above. There will be a core group of snack food items provided, each falling into one of the 6 classes described above. In addition, ‘typical’ breakfast, lunch and dinner items will also be provided. There will be at least 2 items per cell per meal, provided in sufficient quantity to assure subject satisfaction. For example, the following items might be provided for breakfast:

**High Sugar High Complex CHO High Protein**

**High Fat** Choc. Donut Biscuits/Gravy Cheese Omelet

**Low Fat** Pop Tarts Bagel/LF Cr. Cheese Egg White Omelet

The machines are under refrigeration and therefore perishable items can be provided. Moreover, the vending room is equipped with a microwave oven and toaster and so complex meals can be prepared. Finally, efforts will be made to find pre-packaged food items which fit the above categories and which can easily be reheated by the subject. We believe this aspect of the study will provide valuable information about food preferences, especially fat preferences, as well as caloric intake and meal timing.

**2. PROP taste test (day 2) (original study only).** The PROP taste test assesses the response to 6-n-propylthiouracil (54) by asking subjects to rate the bitterness of PROP-impregnated paper on a labeled magnitude scale.

**3.** **Dual energy X-ray absorptiometry (day 2).** DXA analysis provides non-invasive assessment of both skeletal density and soft tissue composition by region with good precision. Total body scans require approximately 5-10 minutes depending on the stature and thickness of the subject and have a precision better than 1% for skeletal densities and 2-3% for soft tissue assessments (e.g., % fat, total grams of lean tissue) (55).

During the procedure, the subject is asked to lie flat and quietly on the scan table since the precision of the measurement can be compromised by subject movement during the scan. Proper subject positioning is also important. Once the proper alignment is determined, the subject's ankles and knees are loosely strapped to maintain the position. When the machine is turned on, a 150 μA current flows through the X-ray tube to produce X-rays. As the scan table arm moves from the top of the subject's head downward towards the subject's feet, the shutter opens and a narrow beam of radiation projects upward through the table top and subject. The technician remains in the room with the subject during the scan. After the scan arm clears the subject's feet, the scanner stops and the source shutter closes. The software then calculates body composition in grams of fat tissue, lean tissue, and percent body fat.

**4. Block food questionnaire (day 2).** The Block 2005 food questionnaire is a 100-item food frequency instrument based on the original Block Health Habits and History Questionnaire (56;57) developed by Block et al. at the National Cancer Institute. It allows flexibility in the foods queried and the nutrients assessed as foods on the questionnaire can be added, deleted or rearranged. As a result, it can provide great specificity in nutrient intakes of individuals. We will use the ‘Scantron’ version of the questionnaire which is easily assessed.

**5. Drewnowski taste test (day 3).** Relative sensory preferences for sweetness and fat will be evaluated using the method described by Drewnowski et al. (58) where both taste perception and hedonic response to varying sugar- and fat-containing solutions are scored. Four solutions of varying fat content (skim milk, whole milk, half-and-half, and cream) are combined with varying concentrations of sugar (0%, 5%, 10%, or 20%, by weight) to provide 16 test samples. Perceptions of sweetness, creaminess and preference are numerically scored on a visual analog scale and samples are ranked in order preferred.

**6. Dietary restraint questionnaire (day 3).** The three-factor eating questionnaire (59) classifies eating behavior among individuals on the basis of restraint, disinhibition, and hunger. This classification of behavior provides a format for the development of obesity treatment strategies. This questionnaire does not require administration by an interviewer.

**7. Gormally binge eating questionnaire (day 3).** The Gormally binge eating scale will be used to assess bingeing behavior (60). This questionnaire can be self-administered. The binge eating scale is designed to describe both behavioral manifestations and feelings/cognitions surrounding a binge episode and can successfully discriminate among persons having no, moderate, or severe binge eating problems.

**8. Body image questionnaire for body shape perception (day 3).** The influence of sociocultural factors on body shape perception, especially among minority and non-white populations, has been the subject of some recent investigation (36;37;61). Knowledge of body image perceptions can provide insight into ideals of healthiness and can facilitate weight loss interventions (62). The figures of Stunkard et al. (63) will be used to assess body shape perceptions in a self-administered questionnaire. See Appendix G. This is called the FRS: figure rating scale

**9. Oral glucose tolerance test (day 4).** After a 10-12-hour overnight fast, an intravenous catheter will be placed in a forearm vein for blood withdrawal after which the subject will ingest 75 grams of glucose over 2 minutes. Blood will be drawn at -15, 0, 30, 60, 120 and 180 minutes for plasma leptin, DNA, insulin and glucose determinations. If a subject is known to have been diagnosed with diabetes in the past 6 months, the oral glucose tolerance test will not be repeated.

**10. 24- hour sedentary energy expenditure using the respiratory chamber (day 5-6).** The respiratory chamber is a comfortable, air-tight room (10 x 11 x 8 ft, net volume 19,000 l) constructed as a large, open-circuit indirect calorimeter. It is furnished with a toilet, sink, hide-a-bed, couch, desk, chair, telephone, color television, and radio. The air temperature is constantly maintained at 75°F by an air-conditioning system. Visual contact with the subject is possible through one of the windows. Food is introduced into the chamber through an air-tight interlock. Spontaneous physical activity is continuously monitored using a radar system based on the Doppler effect. The concentrations of oxygen and carbon dioxide are measured continuously using a paramagnetic oxygen analyzer and an infrared carbon dioxide analyzer. The flow rate of air extracted and replaced by fresh air is measured using a turbine flowmeter. The flow rate and the oxygen and carbon dioxide concentrations of the out flowing air are continuously computed and calculations of oxygen consumption, carbon dioxide production, respiratory quotient, and spontaneous physical activity are made every 15 minutes. The subject will enter the chamber at 7:45 a.m. for 23.5 hours. Urine will be collected over a 24-hour period (day and night collection) for catecholamines and urinary urea. At 7:00 a.m. the following morning, the basal metabolic rate will be determined for 30 minutes using a transparent, ventilated hood, while the subject is awake and lying on the bed in the respiratory chamber. Fresh air will be drawn through the hood to keep the carbon dioxide concentration between 0.5 and 0.8%.

**11. Blood drawing for determination of fasting plasma ghrelin, gut hormone PYY_3-36_, and additional orexigenic/anorexic hormones of interest (days 7-10 or days 7-14).** Research into the causes of obesity has intensified and as a result, new hormones such as ghrelin (64) and gut hormone PYY_3-36_ (65) that regulate appetite and food intake have been uncovered with some regularity. Because this protocol uses very novel methodology, we wish to take blood samples during the vending machine phase of the study to determine levels of these hormones during and after ad libitum food intake in these subjects. Therefore, each morning during days 7-10 (or 7-14), 20 cc of blood will be taken and stored for use in assessing levels of newly discovered hormones.

**12. Additional Psychology and Behavioral questionnaires:**

a. The Questionnaire on Eating and Weight Patterns ‑ Revised (QEWP‑R) (38): a self-report instrument used for diagnosis of binge eating disorder (BED). It is designed to describe both behavioral manifestations and feelings/cognitions surrounding a binge episode and can successfully discriminate among persons having no, moderate, or severe binge eating problems.

1. Night Eating Diagnostic Scale (NEDS) (39): developed to diagnose night eating syndrome.
2. Emotional Appetite Questionnaire (EMAQ): to examine eating response to both positive and negative emotions and situations (40).
3. Perceived Stress Scale (PSS) (41): a 14-item self-reported questionnaire used to assess the stress domains of unpredictability, lack of control, burden overload, and stressful life circumstances in the last 28-days.
4. The Inventory for Depressive Symptomatology (IDS –SR) (42) a measure of depressive signs and symptoms, applicable to atypical depression which has been associated with overeating. Those who report suicidal ideation or have scores consistent with major depression will be immediately interviewed by a trained behavioral health professional and referred for appropriate treatment and follow-up. The clinical research unit employs a licensed psychologist, but the Phoenix Indian Medical Center also has on-call behavioral health professionals available 24 hours/day.
5. Physical Anhedonia Scale (43): a 61-item scale to assess sensitivity to reward (STR). It is designed to reflect the degree to which individuals take pleasure from, and are motivated to engage in, rewarding behaviors. High scores on this scale reflect the anhedonic end of the STR dimension while low scores reflect ‘hedonia’ or the enhanced ability to seek out and enjoy natural rewards.
6. MacArthur Scale of Subjective Social Status (44): should be administered with rest of psych questionnaires and at all time points
7. U.S. Adult Food Security Survey Module: Six-Item Short Form (46;47): This should be administered with the rest of the psych questionnaires at baseline and then at longer term follow-ups, but not at the end of an inpatient admission
8. Computerized Psychological Performance Tests:
   1. Iowa Gambling Task (48;49): to evaluate decision-making. Subjects will be instructed to try to gain as much fake money as possible by drawing 100 selections from a choice of 4 decks of cards, while starting with a loan. The decisions to choose from the decks should become motivated by reward and punishment schedules inherent in the task. Two of the decks are disadvantageous, producing immediate large rewards but these are (after a pre-punishment phase of about 10–15 cards) accompanied by significant money loss due to extreme punishments. The other 2 decks are advantageous; reward is modest but more consistent and punishment is low.
   2. Stroop Color Word Test (50): to assess selective attention. Words are presented on the screen in either a congruent (color screen with words printed in color) or incongruent set (color-word screen where the color and the word do not match [for example, the word “green” is printed in blue ink]. The subject has to either read the words or name the ink colors as quickly as possible within a time limit.
   3. Wisconsin Card Sorting Test (51): to examine cognitive executive functioning. Subjects are presented with 4 stimulus cards and 128 response cards. They are instructed to respond with response cards to match each of the 4 stimulus cards (based on either color, form or number, but directions are ambiguous), and are told if they are right or wrong. Once a certain number of correct answers are made, the sorting principle is changed without warning, requiring the subject to figure out the new sorting strategy.
   4. Go/No Go Task (52): to examine inhibitory control. Subjects are required to press a key whenever a target stimulus is presented and to refrain from pressing the key when a non-target stimulus is presented.

**13.**  **Monitoring of spontaneous physical activity during overfeeding.** The activity monitors will include i) Actiheart - combined HR monitor and uniaxial accelerometer (chest-worn); ii) Actical – omnidirectional accelerometer (wrist, ankle, and/or hip-worn). The combined heart rate and movement monitor (Actiheart) clips on two standard EKG electrodes attached to the chest.

**14. Measurement of core temperature changes during overfeeding.** We will use the CorTemp™ core body temperature monitoring system to assess changes in body temperature with overfeeding. The system uses an easy-to-swallow (0.88” long x 0.42” wide), wireless sensor to transmit a signal to a data recorder worn on the hip. The data recorder can be worn by volunteers for at least 24 hours to provide a continuous measurement of body temperature change. According to the manufacturer, the capsule passes easily in stool and does not need to be collected after the end of the measurement period. Whether or not an active sensor is present in the volunteer can be detected by the data recorder.

**15. Manipulation of dietary macronutrient content.** Study volunteers will be on the standard weight maintenance diet for the first 3 inpatient days followed by a 24 hour stay in the respiratory chamber for the assessment of sedentary energy expenditure and its components, as per protocol. Upon exiting the chamber, the participants will then begin a 3-day period of either an isocaloric (weight maintenance) high fat (50% fat, 30% carbohydrate, 20% protein) or an isocaloric high carbohydrate (60% carbohydrate, 20% fat, and 20% protein) diet. The third day of diet will again be spent in the respiratory chamber on the high fat or high carbohydrate diet at the chamber weight maintenance intake level. Subsequently, study participants will be allowed free access to the vending machines for 3 days.

**16.** **Needle biopsy of abdominal and femoral subcutaneous adipose tissue.** Adipose tissue will be removed by needle biopsy from the abdominal area, usually at McBurney’s point or at the equivalent site on the left side, and from the femoral area, at the anterior-lateral aspect of the thigh, one- to two-thirds of the distance from the superior iliac spine to the patella. After thorough washing and sterilization, local anesthesia of the cutaneous and subcutaneous tissue will be established using 1% lidocaine. The needle biopsy is then performed using a 12 ml plastic syringe and 14-gauge blunt, closed end needle, with an opening on the side. The needle will be repeatedly moved back and forth in the subcutaneous adipose tissue, while maintaining suction on the syringe, until collection of a sufficient amount of sample for study. The adipose tissue is then rapidly exposed to collagenase to obtain isolated cells that can be counted and measured.

**17.­­­ The use of wearable sensors to estimate food intake and mass (day 7; day 1 of vending).**  A subset (n=50) of volunteers will be invited to wear the strain and swallowing sensors. These sensors consist of a strain sensor (a band-aid that attaches below outer ear) that monitors chewing and a microphone (on a neck strap) that detects swallowing. The sensors can be easily worn by individuals of all sizes, and thus can be used in a wide range of populations. The swallowing monitor is completely based on consumer electronics: it is paintball protection collar with a microphone and an MP3 player packaged inside an Altoids box. The chewing monitor is based on commercially available but not consumer electronics: the sensor is a band-aid attached below an ear and the data logger and battery are packaged inside another Altoids box to be carried in a chest pocket. The electronics are powered by a 3V Li-ion battery commonly found in consumer electronics. The results of previous studies (66) showed that metrics derived from chewing and swallowing events can be used to reliably (>95% accuracy) identify each occurrence of food ingestion with time resolution of 30 seconds; differentiate between ingestion of solids and liquids (>91% accuracy) and predict the mass of ingested solids and liquids (>91% solids, >83% liquids). It has also been shown (67;68) that swallowing instances can be automatically identified by a computer algorithm from the data captured by a miniature microphone. The combination of these methods in a miniature wearable device can enable objective diagnostics and monitoring of ingestive behavior and caloric intake.

Volunteers that wear the sensors will be given a calibration meal on their first day of eating from the vending machine. This calibration meal consists of the same foods and amounts (by weight) for each subject and will be observed by a study worker and filmed for further analysis. The meal is necessary when wearing the sensors in order to analyze the collected data. Following the calibration meal, volunteers will continue wearing the sensors for the remainder of the 24 hours while using the vending machine system. Further eating episodes will not be observed beyond standard, vending machine use observation. After the initial 24 hours, the sensors will be removed and volunteers will continue eating from the vending machines for two more days. Data collected from the sensors will be compared to known data on specifics of food intake and time of eating taken from the vending machine to assess the accuracy of the sensors in picking up all the eating episodes and mass of food in a free living situation.

**18. Measurement of dietary fat oxidation while in the respiratory chamber. P**rior to entering the respiratory chamber, subjects will provide a baseline urine sample. A breakfast will then be provided, which will include a 100 ml serving of liquid meal replacer drink (Ensure™) containing a dietary fat tracer (d_31_-palmitate) at 15 mg/kg. Subjects will collect all urine while in the respiratory chamber as part of the normal procedures. A sample of the 24h urine will be analyzed for deuterium content. From this, an estimate of the cumulative oxidation of dietary fat over the prior 24h can be determined (69).

**19. Vending Machine Exit Questionnaire:** This is an 11-item questionnaire designed to assess motivation to eat while using the vending machines. It includes items related to mood, liking of food, and timing of meals.

**Sample size calculations and data analysis.** The sample size is difficult to estimate as there is little data on the genetic influence on food intake in larger populations. Thus, any sample size calculation should be viewed as preliminary. The sample size calculation was based on the mean caloric intake for volunteers who have participated in this protocol to date of (mean ± SD) 4343 ± 1213 kcal/day. Assuming that alleles are in Hardy-Weinberg equilibrium and the action is additive, then we calculate the sample size required to detect an allele that accounts for 2% of the variance at alpha=0.05 and power=0.90 is 521. Differences in food intake by genotype will be analyzed using regression analyses adjusted for co-variates.

The overfeeding arm of the study will recruit an additional 30 individuals, of any ethnic group, for sufficient power to investigate the above associations, since the original ghrelin study was performed with 30 individuals.

For the high carbohydrate and high fat arm of the study, sample size was based on the mean kilocalories per day for volunteers while eating from the vending machines (mean ± SD, 4343±1213 kcal/d) after eucaloric mixed macronutrient diet. Previous data had shown that the intra-class correlation for the mean kilocalories for volunteers who repeated the study was 0.9. The standard deviation for the difference in mean kilocalories between individuals repeating the vending machine study is 542.5**.** Based on previous studies by Eckel et al. (26), an increase in the carbohydrate percentage of the meals from 30% to 60% should results in an approximate 300 kcal increase in the carbohydrate balance. Based on our previous work and the relationship between carbohydrate balance and mean kilocalorie intake, a 300 kcal difference in carbohydrate balance should result in a 510 kcal difference in mean kilocalories during the 3-day vending machine period. Based on these numbers, the calculated sample size needed to detect a 510 kcal difference at a power of 90% and an alpha of 0.05 would be 50 individuals of any ethnic group.

**POSSIBLE RISKS AND HAZARDS**

1. **Radiation exposure due to dual energy X-ray absorptiometry** -The radiation exposure due to DEXA is 2 mrem. A typical radiation dosage from a chest X-ray is 20 mrem. These values can be used to compare the relative risk of a total body scan by DEXA. For subjects that move excessively during the scan, a complete or partial repeat of the scan procedure may be necessary and thus these subjects could receive a maximum of 4 mrem per procedure.

2. **Questionnaires and computerized performance tests** - There are no known risks associated with completing any of these questionnaires or tests. However, as some of the questions may be of a highly personal nature, subjects will be informed that they do not have to respond to all the questions if they choose not to do so. If this is the case, notations to that effect will be noted in the patient’s record.

3. **Taste tests** - There are no known risks to performing these tests.

4. **Vending machines to assess actual fat intake** - There are no known risks to performing this test.

5. **Oral glucose tolerance test** - This test carries the risk of having an indwelling catheter in place for 3 hours. This includes hematoma, ecchymoses and infection.

6. **Twenty-four hour energy expenditure measurement in the respiratory chamber** - There are no known risks associated with this measurement. At any time, the subject can have visual or auditory contact with the nurses. A nurse is also charged with checking the temperature in the room as well as the different parameters listed on our computer screen every 4 hours. In case of any discomfort (cold, claustrophobia), the volunteer can leave the chamber.

7. **Blood drawing for hormone assessment** - This test carries the risk of developing a hematoma at the site of venipuncture.

8. **Actiheart and Actical activity monitors** - The activity monitors will include i) Actiheart - combined HR monitor and uniaxial accelerometer (chest-worn); ii) Actical – omnidirectional accelerometer (wrist, ankle, and/or hip-worn). The combined heart rate and movement monitor (Actiheart) clips on two standard EKG electrodes attached to the chest. This device has no buttons or display and is much lighter (8 grams) than any other movement sensors or heart rate monitors currently used in epidemiological research. The dimensions of the Actical are 28 x 27 x 10 mm, and it weighs 17 grams.

9. **CorTemp™ core body temperature monitoring system** – The system is intended for the measurement of continuous core body temperature. Contraindications for use are as follows:
 a. Volunteers weighing less than 80 lbs (36.4 kg).

b. In the presence of known/suspected obstructive disease of the GI tract, including but not limited to diverticulitis and inflammatory bowel disease.

c. In volunteers exhibiting or having a history of disorders or impairment of the gag reflex.

d. In any volunteer with previous GI surgery.

e. In any volunteer having felinization of the esophagus.

f. In volunteers who might undergo NMR or MRI scanning during the period that the CorTemp Core Body Temperature Sensor is within the body.

g. In volunteers with hypomotility disorders or the GI tract, including but not limited to ileus.

h. In volunteers with a cardiac pacemaker or other implanted electromedical device.

Prior to administering the CorTemp pill, the contra-indications will be reviewed to make sure the volunteer is an appropriate candidate.

10. **Overfeeding** – The overfeeding in this study is short-term (2 periods of 3 days each) and is not out of the realm of “normal overeating”. Evidence for this is the food intake data while on the vending machines during the original study (5), where individuals averaged >165% of their weight maintenance energy requirements without adverse effect.

11. **Needle** **biopsy of abdominal wall and femoral adipose tissue and muscle tissue** – The potential hazards of this procedure are a local hematoma or infection at the site of the biopsy. These hazards are greatly minimized by attention to hemostasis, application of local pressure, and proper use of sterile technique. The biopsies are performed in the tangential plane to the abdominal wall so the risk of abdominal cavity puncture or epigastric artery laceration are essentially nil.

12. **Wearable sensors to estimate food intake and mass –** Except for the inconvenience of wearing the strain sensor and swallowing collar, there is no risk associated with these sensors. The IRB at Clarkson University has previously approved the use of the swallowing monitor that will be used in this study. A stationary version of the chewing sensor has also been approved by the Clarkson IRB. We propose to use a slightly modified version of the sensor that allows for free ambulation by volunteers.

13**. Measurement of dietary fat oxidation while in the respiratory chamber -** Deuterium is a stable (non-radioactive) isotope tracer that is given in minute quantities. There is minimal risk associated with this test (70). Deuterium oxide has been used extensively for the measurement of total body water spaces in man (71;72). The natural abundance of deuterium in tap water is 150 ppm and a tracer dose of 4-5 grams in an adult increases this to about 250 ppm. There have been no reports of any signs of toxicity or abnormalities in metabolism using this tracer amount.

**BENEFIT FOR THE PARTICIPANTS**

There is no benefit to the participant for taking part in this study. However, some of the data derived from this study may help us to define some behaviors that put people at greater risk of obesity and its complications. Individuals exhibiting unhealthy behaviors will be offered feedback and counseling to correct these behaviors.

**RESEARCH USE OF HUMAN SAMPLES, SPECIMENS OR DATA**

Blood (stored as plasma or serum) urine, and fat samples not immediately used for study purposes will be stored for future measurements. Stored samples and specimens will be used only to measure factors that relate to diabetes, obesity and their complications. Stored samples, specimens or data may be sent to collaborators for specific measurements or analyses. All stored samples, specimens, and data will be coded so that when sent for measurements the identity of the volunteer remains confidential. Identification of coded samples will be kept in a secure password-protected database accessible only to investigators, but samples will be identifiable in case specific tests yield clinical information of importance to a particular volunteer or so samples can be destroyed per volunteer request (see below). Samples will be used only for research and not for commercial purposes. Research volunteers will not be informed of individual results from analyses performed specifically for research purposes, unless there is clear evidence accepted by the medical community that these results will impact the volunteer’s individual medical care or future health. Samples will be stored until used unless the volunteer requests in writing that the samples be destroyed. Reports of samples lost due to technical issues or destroyed secondary to volunteer request will be included in the annual renewal report.

At this time, there is a collaboration set up with Matthias H. Tschoep, MD, of the Obesity Research Center at the University of Cincinnati’s Genome Research Institute, in Cincinnati, OH, for measuring hormones that may be related to obesity and food intake.

We are collaborating with Paul Franks, Ph.D., of the Umeå University Hospital, Umeå, Sweden. Dr. Franks will aid in analysis of spontaneous physical activity data as collected by activity monitors in the overfeeding arm of the study. Dr. Franks will receive data from the activity monitors and other metabolic parameters collected during this arm of the study.

We also are collaborating with Dr. Jack Yanovski, Unit on Growth and Obesity, National Institute of Child Health and Disease, Bethesda, MD. Dr. Yanovski will measure brain derived neurotrophic factor (BDNF), which may have a role in energy homeostasis. He will measure BDNF in serum and plasma to examine its relationship with energy expenditure, insulin action, and adiposity.

We are collaborating with Dr. Edward Sazonov, Associate Professor, Department of Electrical and Computer Engineering, Clarkson University, Potsdam, NY. Dr. Sazonov has developed the wearable chew/swallow sensors for energy intake monitoring that some of our volunteers will wear while eating from the vending machine system. Dr. Sazonov is providing the sensors and training as well as assisting with data analysis and interpretation.

We are also collaborating with Daniele Piomelli, Ph.D., University of California, Irvine, Irvine, CA. Dr. Piomelli will measure endocannabinoids, fatty acids, and fatty acid derivatives in plasma samples from research volunteers participating in this protocol. Analyses will test whether these molecules are related to food intake, choice of foods, meal frequency, macronutrient intake and time of food consumption.

**ADVERSE EVENT REPORTING**

Adverse events that occur as the direct result of the testing procedures in this study will be forwarded to the NIDDK IRB. Since this is not a treatment study, these events are expected to be infrequent. Previously undiagnosed medical conditions discovered by laboratory testing or imaging will not be reported as adverse events, but will receive appropriate follow-up and referral to the primary care provider.

**DATA AND SAFETY MONITORING**

Because this is not an intervention trial, data and safety monitoring issues will be limited to events associated with obtaining the measures for this study. The Principal Investigator will, therefore, serve in this capacity and report these events directly to the NIDDK IRB.

**BLOOD DRAWING**

1) Screening lab work 25 ml

2) OGTT (3-hr) 77 ml

3) Blood for hormone assessment (3-day vend – 4 days) 148 ml

**TOTAL** for 3-day vend  **250 ml**

**Blood Drawing For Ghrelin Suppression And Food Intake Substudy**

1) Screening lab work 25 ml

2) OGTT (3-hr) 30 ml

3) Blood for hormone assessment (3-day vend)*2 148 ml

4) Blood for hormone measurements prior to and after 108 ml

overfeeding period and weight maintenance period

**TOTAL** for study  **311 ml**

**Blood Drawing For Effect Of Macronutrient Substudy**

1) Screening lab work 25 ml

2) OGTT (3-hr) 30 ml

3) Blood for lymphocyte transformation 20 ml

4) Blood for hormone assessment (3-day vend)*2 120 ml

5) Blood for hormone assessment prior to high carbohydrate 40 ml

and high fat diets

**TOTAL** for study  **235 ml**

**Blood drawing for follow-up visits for overfeeding and macronutrient studies**

1) Fasting blood draw 30 ml

**PAYMENT**

1) Daily payment $35/day x 10 days $350.00

2) Three inconvenience units for the metabolic chamber 75.00

3) 3 inconvenience units for the questionnaires & performance tests 75.00

4) One inconvenience unit for the OGTT study 25.00

5) Three inconvenience units for the vending machine study 75.00

6) Two inconvenience units for blood drawing 50.00

7) Ten inconvenience units for fat biopsies 250.00

8) One inconvenience unit for measurement dietary fat oxidation 25.00

9) One inconvenience unit for wearing chew/swallow sensors 25.00

**TOTAL $950.00**

BONUS PAYMENT FOR STUDY COMPLETION $100.00

**GRAND TOTAL $1050.00**

**Payment for Ghrelin Suppression and Food Intake Substudy**

1) Daily payment $35/day x 20 days $700.00

2) Three inconvenience units for each metabolic chamber * 2 150.00

3) 3 inconvenience units for the questionnaires & performance tests 75.00

4) One inconvenience unit for the OGTT study 25.00

5) Three inconvenience units for the vending machine study*2 150.00

6) Two inconvenience units for blood drawing*2 100.00

7) One inconvenience unit for Temperature sensor*2 50.00

8) One inconvenience unit for wearing activity monitors 25.00

**TOTAL $1275.00**

BONUS PAYMENT FOR STUDY COMPLETION $100.00

**GRAND TOTAL $1375.00**

**Payment for Effect of Macronutrient Content Substudy**

1) Daily payment $35/day x 21 days $735.00

2) Three inconvenience units for the metabolic chamber*3 225.00

3) 3 inconvenience units for the questionnaires & performance tests 75.00

4) One inconvenience unit for the OGTT study 25.00

5) Three inconvenience units for the vending machine study*2 150.00

6) Two inconvenience units for blood drawing*2 100.00

**TOTAL $1310.00**

BONUS PAYMENT FOR STUDY COMPLETION $100.00

**GRAND TOTAL $1410.00**

**Payment for Each Revisit (Overfeeding and Macronutrient Content Study Arms)**

1) Daily payment $35/day x 1 day $35.00

2) One inconvenience unit for fasting blood work 25.00

**TOTAL $60.00**

References

1. Kuczmarski R, Flegal K, Campbell S, Johnson C. Increasing prevalence of overweight among US adults. JAMA 1994;272:205-11.

2. Rosenbaum M, Leibel R, Hirsch J. Obesity. N Engl J Med 1997;337:396-407.

3. Ravussin E. Metabolic differences and the development of obesity. Metabolism 1995;44:12-4.

4. Friedman M, Brownell K. Psychological correlates of obesity: moving to the next research generation. Psychol Bull 1995;117:3-20.

5. Salbe A, Tschop M, DelParigi A, Venti C, Tataranni P. Negative relationship between fasting plasma ghrelin concentrations and ad libitum food intake. J Clin Endocrinol Metab 2004;89:2951-6.

6. Tschop M, Weyer C, Tataranni P, Devanarayan A, Ravussin E, Heiman M. Circulating ghrelin levels are decreased in human obesity. Diabetes 2001;50:707-9.

7. Ravussin E, Tschop M, Morales S, Bouchard C, Heiman M. Plasma ghrelin concentration and energy balance: overfeeding and negative energy balance studies in twins. J Clin Endocrinol Metab 2001;86:4547-51.

8. Danforth Jr, E, Horton, ES, O'Connell, M, Sims, EA, Burger, AG, Ingbar, SH, Braverman, L, and Vagenakis, AG. Dietary-induced alterations in thyroid hormone metabolism during overnutrition. J.Clin.Invest. 64, 1336-1347. 1979.

9. Burger A, O'Connell M, Scheidegger K, Woo R, Danforth Jr E. Monodeiodination of triiodothyronine and reverse triiodothyronine during low and high calorie diet. J Clin Endocrinol Metab 1987;65:829-35.

10. Ortega, E, Pannacciulli, N, Bogardus, C, and Krakoff, J. Free triiodothyronine (FT3) plasma concentrations predict weight change in euthyroid individuals. Am.J.Clin.Nutr. 85(2), 440-445. 2007.

11. Tataranni, PA, Young, JB, Bogardus, C, and Ravussin, E. A low sympathoadrenal activity is associated with body weight gain and development of central adiposity in Pima Indian men. Obes.Res. 5, 341-347. 1997.

12. Hill A, Rogers P, Blundell J. Techniques for the experimental measurement of human eating behavior and food intake: a practical guide. Int J Obes 1995;19:361-75.

13. de Castro J. Genetic influences on daily intake and meal patterns of humans. Physiol Behav 1993;53:777-82.

14. de Castro J. Independence of genetic influences of body size, daily intake and meal patterns of humans. Physiol Behav 1993;54:633-9.

15. Tepper B. 6-n-propylthiouracil: A genetic marker for taste, with implications for food preferences and dietary habits. Am J Hum Genet 1998;63:1271-6.

16. Astrup A, Buemann B, Western P, Toubro S, Raben A, Christensen N. Obesity as an adaptation to a high-fat diet: evidence from a cross-sectional study. Am J Clin Nutr 1994;59:350-5.

17. Doucet E, Tremblay A. Food intake, energy balance and body weight control. Eur J Clin Nutr 1997;51:846-55.

18. Ravussin E, Tataranni P. Dietary fat and human obesity. JADA 1997;97:S42-S46.

19. Westerterp K, Verboeket-van de Venne W, Westerterp-Plantega M, Velthuis-te Wierik E, de Graaf C, Westrate J. Dietary fat and body fat: an intervention study. Int J Obes 1996;20:1022-6.

20. Heitmann B, Lissner L, Sorenson T, Bengtsson C. Dietary fat intake and weight gain in women genetically predisposed for obesity. Am J Clin Nutr 1995;61:1213-7.

21. Cooling J, Blundell J. Are high-fat and low-fat consumers distinct phenotypes? Differences in the subjective and behavioral response to energy and nutrient challenges. Eur J Clin Nutr 1998;52:193-201.

22. Flegal K, Carroll M, Kuczmarski R, Johnson C. Overweight and obesity in the United States: prevalence and trends, 1960-1994. Int J Obes 1998;22:39-47.

23. Kuzmarski R, Flegal K, Campbell S, Johnson C. Increasing prevalence of overweight among US adults: The National Health adn Nutrition Examination Surveys, 1960-1991. JAMA 1994;272:205-11.

24. Willet W. Is dietary fat a major determinant of body fat? Am J Clin Nutr 1998;67:556S-62S.

25. Pannacciulli N, Salbe A, Ortega E, Venti C, Bogardus C, Krakoff J. The 24-h carbohydrate oxidation rate in a human respiratory chamber predicts ad libitum food intake. Am J Clin Nutr 2007;86:625-32.

26. Eckel, R. H., Hernandex, T. L., Bell, M. L., Weil, K. M., Shepard, T. Y., Grunwald, G. K., Sharp, T. A., Francis, C. C., and Hill, J. O. Carbohydate balance predicts weight and fat gain in adults. Am.J.Clin.Nutr. 83, 803-808. 2006.

27. Flatt J. Macronutrient composition and food selection. Obes Res 2001;9:256S-62S.

28. Champagne C, Bray G, Kurtz A et al. Energy intake and energy expenditure: a controlled study comparing dietitians and non-dietitians. J Am Diet Assoc 2002;102:1428-32.

29. Prentice A, Black A, Murgatroyd P, Goldberg GCW. Metabolism or appetite: questions of energy balance with particular reference to obesity. J Hum Nutr Diet 1989;2:95-104.

30. Weber J, Reid P, Greaves K et al. Validity of self-reported energy intake in lean and obese young women, esing two nutrient databases, compared with total energy expenditure assessed by doubly labeled water. Eur J Clin Nutr 2001;55:940-50.

31. Day N, McKeown N, Wong M, Welch A, Bingham S. Epidemiological assessment of diet: a comparison of a 7-day diary with a food frequency questionnaire using urinary markers of nitrogen, potassium and sodium. Int J Epidemiol 2001;30:309-17.

32. de Castro J. Methodology, correlational analysis, and interpretation of diet diary records of food and fluid intake of free-living humans. Appetite 1994;23:179-92.

33. Wang D, Kogashiwa M, Ohta S, Kira S. Validity and reliability of a dietary assessment method: The application of a digital camera with a mobile phone card attachment. J Nutr Sci Vitaminol 2002;48:498-504.

34. Wang D, Kogashiwa M, Kira S. Development of a new instrument for evaluating individuals' dietary intake. J Am Diet Assoc 2006;106:1588-93.

35. Kaczkowski C, Jones P, Feng J, Bayley H. Four-day multimedia diet records underestimate energy needs in middle-aged and elderly women as determined by doubly-labeled water. J Nutr 2000;2000:802-5.

36. Oliveria S, Ellison R, Moore L, Gillman M, Garrahie E, Singer M. Parent-child relationships in nutrient intake: the Framingham Children's Study. Am J Clin Nutr 1992;56:593-8.

37. Fisher J, Birch L. Fat preferences and fat consumption of 3- to 5-year-old children are related to parental adiposity. JADA 1995;95:759-64.

38. Spitzer R, Devlin M, Walsh B, Hasin D. Binge eating disorder: a multisite field trial of the diagnostic criteria. Int J Eat Disord 1992;11:191-203.

39. Gluck, ME, Geliebter, A, and Satov, T. Night eating syndrome is associated with depression, low self-esteem, reduced daytime hunger, and less weight loss in obese outpatients. Obes.Res. 9, 264-267. 2001.

40. Geliebter, A and Aversa, A. Emotional eating in overweight, normal weight, and underweight individuals. Eating Behaviors 3, 341-347. 2003.

41. Cohen, S, Kamarck, T, and Mermelstein, R. A global measure of perceived stress. J Health Soc Behav 24, 385-396. 1983.

42. Rush, AJ, Giles, DE, Schlesser, MA, Fulton, CL, Weissenburger, J, and Burns, C. The Inventory for Depressive Symptomology (IDS): preliminary findings. Psychiatry Res. 18, 65-87. 1986.

43. Chapman, L, Chapman, J, and Raulin, ML. Scales for physical and social anhedonia. J.Abnorm.Psychol. 85, 374-382. 1976.

44. Adler N, Epel E, Castellazzo G, Ickovics J. Relationship of subjective and objective social status with psychological and physiological functioning: preliminary data in healthy white women. Health Psychol 2000;19:586-92.

45. Goodman E, Adler N, Kawachi I, Frazier A, Huang B, Colditz G. Adolescents' perceptions of social status: development and evaluation of a new indicator. Pediatrics 2001;108:E31.

46. Bickel, G, Nord, M, Price, C, Hamilton, W, and Cook, J. Measuring food security in the United States. Guide to measuring household food security, revised 2000. 2000. Alexandria, VA, Food and Nutrition Service; Office of Analysis, Nutrition, and Evaluation; U.S. Department of Agriculture.

47. Blumberg S, Bialostosky K, Hamilton W, Briefel R. The effectiveness of a short form of the Household Food Security Scale. Am J Public Health 1999;89:1231-4.

48. Bechara, A, Damasio, AR, Damasio, H, and Anderson, SW. Insensitivity to future consequences following damage to human prefrontal cortex. Cognition 50, 7-15. 1994.

49. Bechara, A, Damasio, H, Tranel, D, and Damasio, AR. Deciding advantageously before knowing the advantageous strategy. Science 275, 1293-1295. 1997.

Ref Type: Generic

50. Stroop, JR. Studies of interference in serial verbal reactions. J.Exper.Psychol. 643-662. 1935.

51. Kongs, SK, Thompson, LL, Iverson, GL, and Heaton, RK. Wisconsin Card Sorting Test - 64 card version. 2000. Odessa, FL, Psychological Assessment Resources, Inc.

52. Murphy F, Sahakian B, Rubinsztein J et al. Emotional bias and inhibitory control processes in mania and depression. Psychol Med 1999;29:1307-21.

53. Geiselman P, Anderson A, Dowdy M, Redmann S, Smith S. Reliability and validity of a macronutrient self-selection paradigm and a food preference questionnaire. Physiol Behav 1998;63:919-28.

54. Bartoshuk, LM, Duffy, VB, and Miller, IJ. PTC/PROP tasting: anatomy, pyschophysics, and sex effects. Physiol.Behav. 56, 165-1171. 1994.

55. Mazess R, Barden H, Bizek J, Hanson J. Dual-energy X-ray absorptiometry for total body and regional bone-mineral and soft-tissue composition. Am J Clin Nutr 1990;55:950-4.

56. Block G, Coyle L, Hartman A, Scoppa S. Revision of dietary analysis software for the Health Habits and History Questionnaire. Am J Epidemiology 1994;139:1190-6.

57. Block G. A review of validations of dietary assessment methods. Am J Epidemiology 1982;115:492-505.

58. Drewnowski, A and Greenwood, MR. Cream and sugar: human preferences for high-fat foods. Physiol.Behav. 30, 629-633. 1983.

59. Stunkard, AJ and Messick, S. The three-factor eating questionnaire to measure dietary restraint, disinhibition and hunger. J.Psychosom.Res. 29, 71-83. 1985.

60. Gormally, J, Black, S, Daston, S, and Rardin, D. The assessment of binge eating severity among obese persons. Addict.Behav. 7, 47-55. 1982.

61. Furnham A, Baguma P. Cross-cultural differences in the evaluation of male and female body shapes. Int J Eat Disord 1994;15:81-9.

62. Kumanyika S. Special issues regarding obesity in minority populations. Ann Int Med 1993;119:650-4.

63. Stunkard A, Sorensen T, Schulsinger F. Use of the Danish Adoption Register for the study of obesity and thinness. Res Publ Assoc Res Nerv Ment Dis. 1983;60:115-20.

64. Nakazato M, Murakami N, Date Y et al. A role for ghrelin in the central regulation of feeding. Nature 2001;409:194-8.

65. Batterham, RL, Cohen, MA, Ellis, SM, Le Roux, CW, Withers, DJ, Frost, GS, Ghatei, MA, and Bloom, SR. Inhibition of food intake in obese subjects by peptide YY3-36. N.Engl.J.Med. 349, 941-948. 2003.

66. Sazonov E, Schuckers S, Lopez-Meyer P et al. Toward objective monitoring of ingestive behavior in free living population. Obesity 2009;17:1971-5.

67. Makeyev O, Sazonov E, Schuckers S et al. Recognition of swallowing sounds using time-frequency decomposition and limited receptive area neural classifier. Applications and Innovations in Intelligent Systems XVI: Proceedings of AI-2008m tge 28th SGAI International Conference on Innovative Techniques and Applications of Artificial Intelligence. Cambridge, UK: Springer 2008:33-46.

68. Sazonov E, Makeyev O, Schuckers S, Lopez-Meyer P, Melanson E, Neuman M. Automatic detection of swallowing events by acoustical means for applications of monitoring ingestive behavior. IEE Transactions on Biomedical Engineering 2010;57:626-33.

69. Votruba S, Zeddun S, Schoeller D. Validation of deuterium labeled fatty acids for the measurement of dietary fat oxidation: a method for measuring fat oxidation in free-living subjects. Int J Obes 2001;25:1240-5.

70. Halliday D, Miller A. Precise measurement of total body water using trace quantities of deuterium oxide. Biomed Mass Spectrom 1979;4:82-7.

71. Schoeller D.A., van Santen E. Measurement of energy expenditure in humans by doubly labeled water method. Journal of Applied Physiology 1982;53:955-9.

72. Schoeller D, van-Santen E, Peterson D, Dietz W, Jaspan J, Klein P. Total body water measurement in humans with 18O and 2H labeled water. Am J Clin Nutr 1980;33:2686-93.
